# The association of nocturnal hypoxemia with dyslipidemia in sleep-disordered breathing population of Chinese community: a cross-sectional study

**DOI:** 10.1186/s12944-023-01919-8

**Published:** 2023-09-26

**Authors:** Tong Feng, Guangliang Shan, Huijing He, Guo Pei, Jiaoying Tan, Bing Lu, Qiong Ou

**Affiliations:** 1 The Second School of Clinical Medicine, Southern Medical University, Guangzhou, China; 2 Sleep Center, Department of Respiratory and Critical Care MedicineGuangdong Provincial People’s Hospital (Guangdong Academy of Medical Sciences)Yuexiu District, Southern Medical University, No.106 Zhongshan Road, Guangzhou City, Guangdong Province, China; 3 Department of Epidemiology & Biostatistics, Institute of Basic Medical Sciences, Chinese Academy of Medical Sciences/School of Basic Medicine, Peking Union Medical College, Beijing, People’s Republic of China

**Keywords:** Hypoxemia, Visceral obesity, Sleep-disordered breathing, Community

## Abstract

**Background:**

Currently, there is limited and controversial clinical research on the correlation between sleep-disordered breathing (SDB) and dyslipidemia. This discrepancy in findings may be because studies that primarily focused on hospital-based populations may not be applicable to community-based populations. Therefore, the primary objective of this research endeavor is to scrutinize the correlation between nocturnal hypoxemia and blood lipid concentrations among adult individuals residing in the community who exhibit symptoms of SDB. Additionally, this study aimed to identify the nocturnal hypoxia parameters having the strongest correlation with this relationship.

**Methods:**

This cross-sectional study collected data from The Guangdong Sleep Health Study, which included 3829 participants. Type IV sleep monitoring was employed to measure hypoxemia parameters, and lipoproteins were evaluated using fasting blood samples. To understand the association between dyslipidemia and hypoxemia parameters, a multivariable logistic regression model was used. Subgroup analyses were conducted to stratify data according to age, sex, waist circumference, and chronic diseases.

**Results:**

The age of the individuals involved in the study spanned from 20 to 90 years. The average age of the participants was 56.15 ± 13.11 years. Of the total sample size, 55.7% were male. In the fully adjusted model, the meanSpO2 was negatively associated with hyperlipidemia (0.9303 [95% confidence interval 0.8719, 0.9925]). Upon conducting a nonlinearity test, the relationship between the meanSpO2 and hyperlipidemia was nonlinear. The inflection points were determined to be 95. When meanSpO2 ≥ 95%, a difference of 1 in the meanSpO2 corresponded to a 0.07 difference in the risk of hyperlipidemia.

**Conclusions:**

This study revealed that higher meanSpO2 is significantly and negatively associated with hyperlipidemia in adult community residents with SDB, particularly when the meanSpO2 exceeds 95. This finding emphasizes the importance of close monitoring for dyslipidemia, which is considered an early indicator of atherosclerosis in patients with SDB who experience nocturnal hypoxia.

**Supplementary Information:**

The online version contains supplementary material available at 10.1186/s12944-023-01919-8.

## Background

Sleep-disordered breathing (SDB) is a prevalent condition affecting many individuals worldwide. It is characterized by the frequent occurrence of episodes involving apnea, which is the temporary cessation of breathing, and hypopnea, which is decreased airflow. These episodes can result in a lack of oxygen during sleep. Notably, recent investigations indicate that approximately 936 million adults are affected by SDB. It is worth noting that among different countries, China holds the distinction of having the highest prevalence rate of SDB [1]. The prevalence of SDB is expected to increase in the future because of the increasing prevalence of obesity and aging [2]. Unfortunately, SDB is often undiagnosed in clinical settings [3].

Multiple studies have provided compelling evidence that there exists a robust association between SDB and the development of cardiovascular disease [4]. If left untreated, patients with SDB face a substantially heightened susceptibility to encountering complications related to the heart and cerebrovascular system, as well as increased mortality from all causes [5]. This increased cardiovascular risk is attributed to a reduction in oxygen levels, which activate the sympathetic nervous system and lead to elevated oxidative stress and inflammation [6–8]. One potential explanation for the connection between SDB and cardiovascular disease is that existing treatments may not completely reverse the damage that has already occurred. Therefore, early detection and intervention of SDB are essential to minimize the adverse consequences of cardiovascular disease. Additionally, SDB-induced hypoxia plays a role in adipose tissue dysfunction, release of pro-inflammatory molecules called lipokines, activation of the sympathetic nervous system, and regulation of hormone-sensitive lipases, which can contribute to lipolysis [9].

Currently, clinical investigations into the relationship between SDB and dyslipidemia are limited and controversial. The variability in the results may be due to differences in study samples, and findings from hospital-based studies may not be applicable to community-based populations. A study in Spain showed that lower levels of mean nocturnal oxygen saturation (meanSpO2) were associated with dyslipidemia in a sample of 809 adults [10]. A clinical study in Brazil examined the correlation between total cholesterol (TC), triglycerides (TG), and meanSpO2 in adults aged 18–65 years and revealed a negative correlation between these variables [11]. Similarly, a clinical sample study in France found that higher levels of oxygen desaturation index (ODI) were linked to elevated levels of serum triglycerides among participants [12]. However, among adult males in the general population of Australia, no significant association has been found between hypoxia parameters and lipid measures [13].

Currently, community-based research on SDB is lacking. However, the use of a Type IV sleep monitor could be a valuable tool for screening of SDB in the general population residing in communities, as it allows for easy measurement of nocturnal hypoxemia. By implementing community-based screening, asymptomatic SDB and the associated cardiovascular risks can be detected early. This study investigated the correlation between nocturnal hypoxemia and blood lipid levels in adult community residents with SDB. Additionally, this study aimed to determine the parameters of nocturnal hypoxia that display the strongest correlation with lipid levels. This study is important for informing public health initiatives that target the early detection and management of cardiovascular risks in individuals with SDB. It is worth noting that obesity is a significant confounding factor that should be considered when examining the association between SDB and blood lipids [14, 15]. To address this potential confounding effect, the mediating effect of waist circumference on this relationship. Moreover, a subgroup analysis stratified by waist circumference was conducted to further explore potential correlations.

Due to the limited availability of community-based research, the objective of this study was to establish a correlation between nighttime hypoxemia and serum lipid levels in individuals residing in the community. It is imperative to conduct community-based studies in order to identify unconventional or asymptomatic SDB in individuals who are commonly perceived to have a low risk of SDB. The current study hypothesis was that blood oxygen parameters are connected to blood lipid parameters in patients with SDB and that obesity may influence this relationship.

## Methods

### Study participants

This cross-sectional study, known as the Guangdong Sleep Health Study, was conducted across multiple sites by the Institute of Basic Medicine of the Chinese Academy of Medical Sciences and the Guangdong Provincial People's Hospital. The research methodology implemented in this study was built upon a previously published study [16]. Data were collected over a six-week period from April 9 to May 18, 2021, and participants were recruited from community settings in two cities, Shantou and Meizhou. This study involved five components: a questionnaire, physical examination, blood tests, bone mineral density analysis, and screening for sleep apnea. Through a rigorous screening process, 1052 participants were selected from an initial pool of 3829 patients who underwent effective sleep monitoring. The selected participants comprised the final dataset used in the analysis.

Exclusion criteria were applied to the participants according to the following factors: (1) absence of sleep-disordered breathing (*n* = 2651); (2) lack of lipoprotein data (*n* = 34); (3) use of lipid-lowering drugs (*n* = 44); (4) taking sedative hypnotic drugs (*n* = 26); and (5) previous treatment for sleep apnea (*n* = 22).

The study protocol was approved by the Ethics Committee of Guangdong Provincial People's Hospital (GDREC2020221H) and all participants provided informed consent prior to their involvement in the study.

### Nocturnal blood oxygen monitoring

The study employed a trustworthy type-IV sleep-monitoring device (Figures S1 and S2) developed by Chengdu CloudCare Healthcare Co. Ltd. to monitor the sleep patterns of the participants [17]. The device recorded various parameters during the monitoring process, including 3% or 4% ODI, meanSpO2, lowest level of oxygen saturation observed during sleep (minSpO2), and time of oxygen saturation less than 90% (T90). A significant ODI value of ≥ 7.0 events/h was considered as an indication of the presence of SDB.

### Physical examination

During the physical examination, several measurements were obtained, including height, weight, and blood pressure. The height of the individuals was measured with great precision, ensuring accuracy to the nearest 0.1 cm. In order to determine their body weight, a Tanita bc-420 model body composition analyzer from Japan was utilized. For blood pressure measurements, a digital blood pressure meter (Omron HEM-907, Japan) was used. Trained investigators measured blood pressure three times and subsequently averaged the results. Participants were instructed to rest for 5–10 min prior to blood pressure measurement, refraining from any exercise, alcohol consumption, smoking, or drinking tea for at least 30 min beforehand.

### Blood index

Blood samples were obtained from every individual following an 8-h period of fasting. The collection, transportation, processing, storage, and analysis of the blood samples were all carried out in accordance with established protocols that have been documented in previous publications [16].

### Structural questionnaire

Each participant completed a comprehensive questionnaire that gathered information on various factors, including age, sex, smoking habits, alcohol consumption, and frequency of physical exercise (ranging from 5–7 to days per week to never exercising). Additionally, participants were asked about their medical history, specifically regarding conditions such as hypertension, diabetes, and cardiovascular disease. Individuals were classified as alcohol drinkers if they consumed an average of 50 g or more of alcohol per day for a minimum of one year. However, those who only occasionally drank or consumed a small amount of alcohol during festive occasions such as the Spring Festival were not classified as regular drinkers. The amount of alcohol consumed in grams was calculated by multiplying the volume of alcohol consumed in milliliters by the alcohol content in percentage, and then multiplying the product by 0.8. Individuals were classified as smokers if they smoked an average of one or more cigarettes per day for a minimum of six months. The threshold for consideration as a smoker was set at 150 cigarettes smoked in total within the specified timeframe, and any participant who met any of the above conditions for alcohol consumption or smoking habits was categorized accordingly in the study.

### Variables

In this study, nocturnal blood oxygen parameters were considered as independent variables, specifically ODI, minSpO2, meanSpO2, T90, and T90%. In contrast, dyslipidemia was the dependent variable, which was defined by TC ≥ 6.2 mmol/L, low-density lipoprotein cholesterol(LDL-C) ≥ 4.1 mmol/L, high-density lipoprotein cholesterol (HDL-C) < 1.0 mmol/L, or TG ≥ 2.3 mmol/L [18].

Several covariates were included in this study, and both continuous and grouped variables were considered. The continuous variables comprised age (in years), AST (in μmol/l), fasting blood glucose (in μmol/l), and creatinine (in μmol/l). The grouped variables included sex (male, female), waistline categories (< 95 cm, 95–100 cm, > 100 cm), education level (lower than high school, higher than high school), presence of diabetes (yes, no), presence of hypertension (yes, no), smoking status (present, past, none), drinking status (present, past, none), marital status (single, married, divorced, widowed), and frequency of physical exercise (ranging from 5–7 days per week to never exercising).

The waistline categories were defined as follows: < 95 cm indicated a healthy waistline, 95–100 cm suggested an increased risk of health complications, and > 100 cm indicated a high-risk waistline. Physical exercise was defined as the frequency of exercise per week or month. Hypertension is typically characterized by the administration of antihypertensive medications and/or a systolic/diastolic blood pressure equal to or exceeding 140 mmHg/90 mmHg, respectively [19]. Diabetes is specifically characterized by a fasting blood glucose level equal to or exceeding 7 mmol/L, or the utilization of drugs designated for the management of diabetes, such as oral hypoglycemic medications and/or insulin [20]. Previously published literature provides additional details regarding demographic data, physical examination data, the process of measuring lipoprotein levels, and the collection of other covariates [16].

### Statistical analyses and missing data

Continuous variables are typically represented by their mean plus or minus the standard deviation, illustrating both the central tendency and variability of the data. On the other hand, categorical variables are typically depicted by their frequencies and percentages, providing a breakdown of the data into distinct categories and showing the relative proportions of each category. In the analysis, various statistical tests were used depending on the data distribution. The chi-square test was used for categorical variables, while the Student’s t-test was used for variables that followed a normal distribution. For variables with a skewed distribution, the Mann–Whitney U test was utilized to explore the differences between the groups with and without hyperlipidemia. To assess the association between nocturnal hypoxia parameters and hyperlipidemia, univariate and multivariate logistic regression models were used. These models were built using a stepwise approach, with three distinct models.

For missing data, a comprehensive list of each variable is presented in the Supplementary Material (Table S1). A comparative sensitivity analysis was conducted to address the issue of missing dependent variables. This analysis aimed to determine whether there were any differences between the participants with and without known lipoprotein data. This study aimed to assess whether missing lipoprotein data affected the validity of this study’s findings [21]. Comparative analysis revealed that most variables were similar between the two groups, indicating that missing lipoprotein data were not significantly biased (Table S2).

To address the missing covariates in this study, multiple imputation method using chained equations was employed. This approach was used to mitigate potential biases caused by selection bias or unavailable information. By imputing the missing covariate data, multiple complete sets of data were created, ensuring that the analysis was based on a representative sample that accounted for any missing values [22]. Rubin’s rules were employed to combine the results obtained from the analysis of five imputed datasets [23]. These results indicate that there was no significant difference between the complete dataset and raw data, as shown in Table S3.

Step 1: Collinear diagnostics were conducted to screen for covariates and eliminate any possible multicollinearity between variables. Specifically, the factors with variance expansion coefficients exceeding 10 were discarded, as shown in Table S4. Additionally, the covariances were adjusted if they resulted in a change of at least 10% in the matched odds ratio when added to the model or if they were significantly associated with hyperlipidemia, as shown in Tables S5 and S6 [24]. Three versions of these models were developed. Model 1, the non-adjusted model, did not include covariates. Model 2, the minimally adjusted model, was adjusted only for sociodemographic variables. Finally, Model 3, the fully adjusted model, involved adjusting for all covariates.

Step 2: Due to concerns about the limitations of the logistic regression model in handling nonlinear models, a Generalized Additive Model and smooth curve-fitting technique were employed to address the potential nonlinearity between meanSpO2 and hyperlipidemia. When nonlinearity was detected, a recursive algorithm was employed to calculate the inflection points. Subsequently, a two-piecewise binary logistic regression model was constructed.

Step 3: Subgroup analyses were conducted using a stratified binary logistic regression model. A likelihood ratio test was used to investigate the effect of modification of the subgroup indicators. A mediation study was conducted to determine the extent to which waist circumference mediated the influence of meanSpO2 on hyperlipidemia. The analysis of the mediation effect has employed three equations to examine the relationship between the independent variable (meanSpO2), the mediator variable (waist circumference), and the dependent variable (dyslipidaemia). In order to control for potential confounding factors, variables such as age, sex, education level, marital status, physical exercise, cigarette smoking, alcohol use, diabetes, hypertension, AST, fasting blood glucose, and creatinine were included in the equation. The mediation proportion was utilized to assess the extent of the mediation effect in this particular study.

A sensitivity study was carried out with the aim of guaranteeing the resilience and accuracy of the data analysis process. The meanSpO2 was categorized into quartiles and *P* values were subsequently calculated for trend analysis. This was done to confirm the study findings regarding the meanSpO2 as a continuous variable and to explore any potential nonlinearities.

For statistical analyses, the R software package (version 4.1.2) and EmpowerStats (X&Y Solutions, Inc, Boston, MA) were used. *P*-values < 0.05 were considered statistically significant.

## Results

### Baseline characteristics of the participants

A flow chart representing the process of research registration is shown in Figure S3. Table S8 shown baseline characteristics of the participants with and without sleep-disordered breathing and Table 1 highlights the distribution of clinical characteristics among 1052 individuals with SDB residing in a community. The participants' age varied from 20–90 years, with an average age of 56.15 ± 13.11 years. Of the total sample size, 55.7% were male. It is worth mentioning that individuals diagnosed with hyperlipidemia were comparatively younger and exhibited lower meanSpO2 values when compared to those without hyperlipidemia. No significant differences were observed in the distribution of the ODI, minSpO2, or T90 between the two groups.

**Table 1:** Baseline characteristics of participants

Characteristics	All Participants (*n* = 1052)	With Dyslipidaemia (*n* = 560)	Without Dyslipidaemia (*n* = 492)	*P*-value
Age (years)	56.15 ± 13.11	54.99 ± 13.97	57.47 ± 11.95	0.002
Sex (%)				0.004
Female	466 (44.30%)	225 (40.18%)	241 (48.98%)	
Male	586 (55.70%)	335 (59.82%)	251 (51.02%)	
Martial Status (%)				0.042
Single	43 (4.09%)	31 (5.54%)	12 (2.44%)	
Married	929 (88.31%)	490 (87.50%)	439 (89.23%)	
Divorce	12 (1.14%)	8 (1.43%)	4 (0.81%)	
Widowed	67 (6.37%)	30 (5.36%)	37 (7.52%)	
Education (%)				0.171
Less than high school	499 (47.43%)	253 (45.18%)	246 (50.00%)	
High school	292 (27.76%)	156 (27.86%)	136 (27.64%)	
More than high school	261 (24.81%)	151 (26.96%)	110 (22.36%)	
Waist circumference(%)				0.124
< 95 cm	839 (79.75%)	461 (82.32%)	378 (76.83%)	
95-100 cm	98 (9.32%)	43 (7.68%)	55 (11.18%)	
> 100 cm	79 (7.51%)	40 (7.14%)	39 (7.93%)	
Physical exercise (%)				0.524
5–7 days per week	588 (55.89%)	316 (56.43%)	272 (55.28%)	
3–4 days per week	101 (9.60%)	57 (10.18%)	44 (8.94%)	
1–2 days per week	108 (10.27%)	61 (10.89%)	47 (9.55%)	
≤ 3 days per month	77 (7.32%)	35 (6.25%)	42 (8.54%)	
never exercising	177 (16.83%)	91 (16.25%)	86 (17.48%)	
Cigarette smoking(%)				< 0.001
No	805 (76.52%)	449 (80.18%)	356 (72.36%)	
Former	66 (6.27%)	38 (6.79%)	28 (5.69%)	
Current	181 (17.21%)	73 (13.04%)	108 (21.95%)	
Alcohol use (%)				0.102
No	818 (77.76%)	447 (79.82%)	371 (75.41%)	
Former	23 (2.19%)	14 (2.50%)	9 (1.83%)	
Current	211 (20.06%)	99 (17.68%)	112 (22.76%)	
Diabetes(%)				0.141
No	905 (86.03%)	490 (87.50%)	415 (84.35%)	
Yes	147 (13.97%)	70 (12.50%)	77 (15.65%)	
Hypertension(%)				0.003
No	640 (60.84%)	364 (65.00%)	276 (56.10%)	
Yes	412 (39.16%)	196 (35.00%)	216 (43.90%)	
MeanSpO2	95.42 ± 2.02	95.59 ± 1.96	95.23 ± 2.06	0.004
MinSpO2	81.85 ± 4.96	81.95 ± 4.80	81.75 ± 5.14	0.514
T90%	5.06 ± 10.32	4.98 ± 9.98	5.16 ± 10.71	0.789
T90(s)	1060.07 ± 2053.64	1039.48 ± 1979.30	1083.51 ± 2136.89	0.729
ODI( events/h)	13.80 ± 7.59	13.46 ± 7.16	14.18 ± 8.04	0.122
Total cholesterol (mg/dL)	5.65 ± 1.07	5.21 ± 0.67	6.16 ± 1.20	< 0.001
HDL-C (mg/dL)	1.34 ± 0.38	1.45 ± 0.33	1.21 ± 0.40	< 0.001
LDL-C (mg/dL)	3.30 ± 0.87	3.01 ± 0.57	3.64 ± 1.01	< 0.001
Triglycerides (mg/dL)	1.66 ± 1.25	1.14 ± 0.41	2.25 ± 1.58	< 0.001
AST (U/L)	24.78 ± 10.72	24.19 ± 11.69	25.45 ± 9.47	0.057
Fasting blood glucose(mmol/L)	6.00 ± 1.45	5.88 ± 1.25	6.13 ± 1.65	0.006
Creatinine(umol/l)	74.66 ± 22.75	72.93 ± 23.25	76.63 ± 22.02	0.008

Mean ± SD for continuous variables. Percent for categorical variables

*Abbreviations: ODI* oxygen desaturation index, *MinSpO2* lowest nocturnal oxygen saturation, *MeanSpO2* nocturnal mean oxygen saturation, *T90* night time spent with an oxygen saturation below 90%

### Nocturnal hypoxemia and hyperlipidemia

The objective of this study was to investigate various markers of nocturnal hypoxia, including ODI, minSpO2, meanSpO2, T90, and T90%, and to determine their sensitivity for predicting hyperlipidemia (Table 2). These markers were initially incorporated into a logistic model without adjusting for the covariates. The results indicated that for each 1 percentage increase in meanSpO2, there was a 7% decrease in likelihood of developing of hyperlipidemia, as indicated by an odds ratio (OR) of 0.93 and a 95% confidence interval (CI) of 0.87–0.99 (*P* < 0.001). In the model with minimal adjustments, the OR trends of the odds ratio remained unchanged. Once fully adjusted, there was no significant change in the odds ratio. These consistent findings, regardless of the adjustment strategies employed, indicate that meanSpO2 is an independent risk factor for hyperlipidemia; therefore, meanSpO2 was further categorized into quartiles for the purpose of conducting a sensitivity analysis. The current study results consistently demonstrated a significant trend (*P* < 0.01). Waist circumference may not accurately reflect noncentral body fat deposition, which tends to be more common in females [25]. BMI was included in this study’s adjusted analyses, and the results consistently supported the study findings (Table S9). The relationship between specific lipid profile parameters and oxygen desaturation parameters was examined. The present study revealed a negative correlation between minSpO2 and HDL-C levels (Table S10, S11).

**Table 2:** Odds ratios and 95% confidence intervals of dyslipidaemia among patients with SDB, according to sleep parameters extracted from type IV sleep monitoring

	Model 1	Model 2	Model 3
ODI	1.0127 (0.9966, 1.0290)	1.0086 (0.9922, 1.0254)	1.0087 (0.9915, 1.0263)
MeanSpO2	**0.9129 (0.8566, 0.9728)**	**0.9283 (0.8714, 0.9888)**	**0.9303 (0.8719, 0.9925)**
MinSpO2	0.9919 (0.9680, 1.0164)	0.9938 (0.9693, 1.0189)	0.9953 (0.9702, 1.0210)
T90%	1.0016 (0.9899, 1.0134)	1.0003 (0.9884, 1.0124)	1.0009 (0.9888, 1.0132)
T90	1.0000 (1.0000, 1.0001)	1.0000 (0.9999, 1.0001)	1.0000 (0.9999, 1.0001)
MeanSpO2 quartile			
Q1	ref	ref	ref
Q2	0.8587 (0.6098, 1.2092)	0.8938 (0.6317, 1.2648)	0.9005 (0.6329, 1.2814)
Q3	0.7602 (0.5396, 1.0708)	0.8293 (0.5844, 1.1767)	0.8657 (0.6054, 1.2378)
Q4	0.5056 (0.3568, 0.7164)	0.5727 (0.3986, 0.8229)	0.5789 (0.3986, 0.8407)
P for trend	0.001	0.004	0.009

Model 1: no covariates were adjusted

Model 2: only sociodemographic variables were adjusted (age, sex, education level, marital status)

Model 3: all covariates presented in Table 1 were adjusted

*Abbreviations: ODI* oxygen desaturation index, *MinSpO2* lowest nocturnal oxygen saturation, *MeanSpO2* nocturnal mean oxygen saturation, *T90* night time spent with an oxygen saturation below 90%, *T90%* percentage of night time with oxygen saturation below 90%

**Fig. 1:**
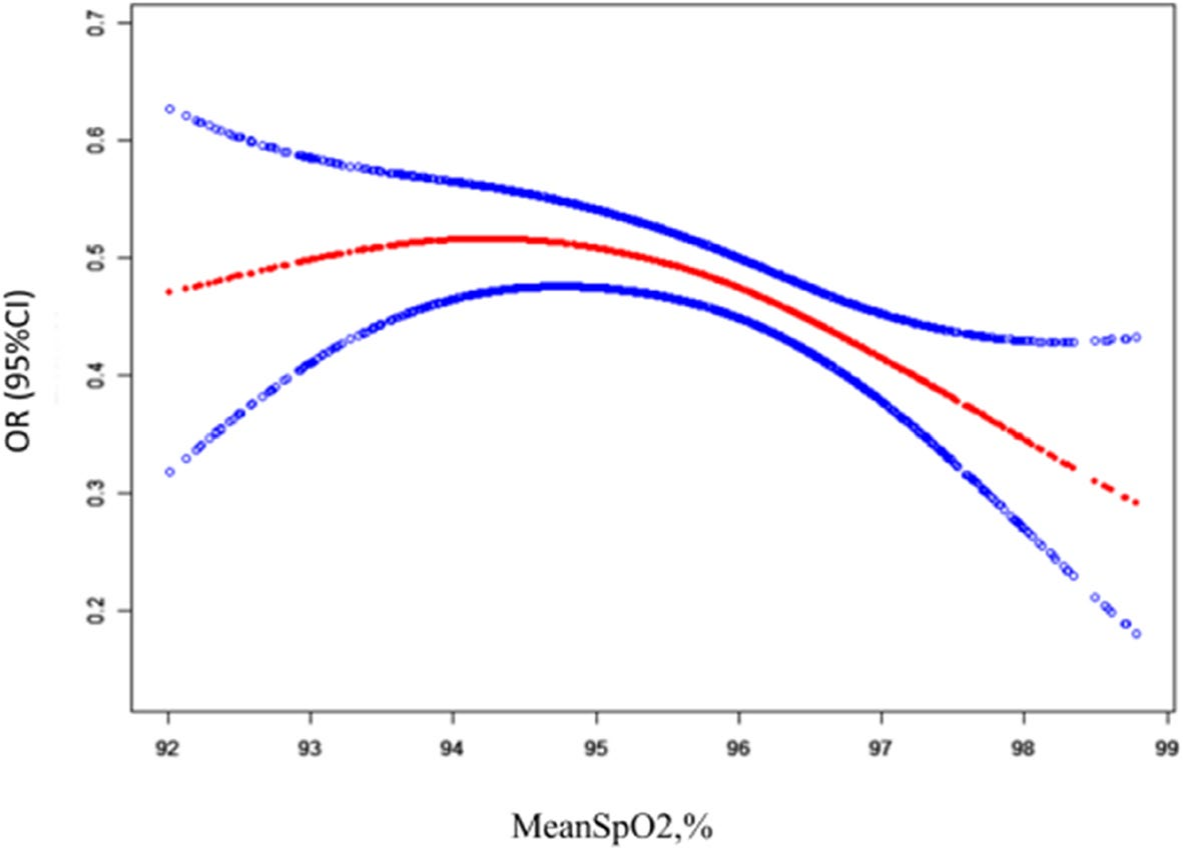
The non-linear relationship between MeanSpO2 and Dyslipidaemia (Models adjusted for all covariates presented Table 1)

Additionally, non-equidistant variations in the odds ratio suggested a potential nonlinear relationship between the meanSpO2 and hyperlipidemia (Fig. 1). The inflection points were identified by implementing a two-piecewise linear regression model (Table 3). A sensitivity analysis revealed that nonlinear trends were similar before and after imputation (Figure S4).

**Table 3:** The results of two-piecewise linear regression model

	MeanSpO2
Fitting model by standard linear regression	0.9334 (0.8747, 0.9961)
Fitting model by two-piecewise linear regression	
Inflection point of MeanSpO2	95
≤ inflection point	1.0053 (0.9186, 1.1001)
> inflection point	0.7964 (0.6835, 0.9281)
P for log likelihood ratio test	0.024

Models adjusted for all covariates presented Table 1

### Subgroup analysis

**Fig. 2:**
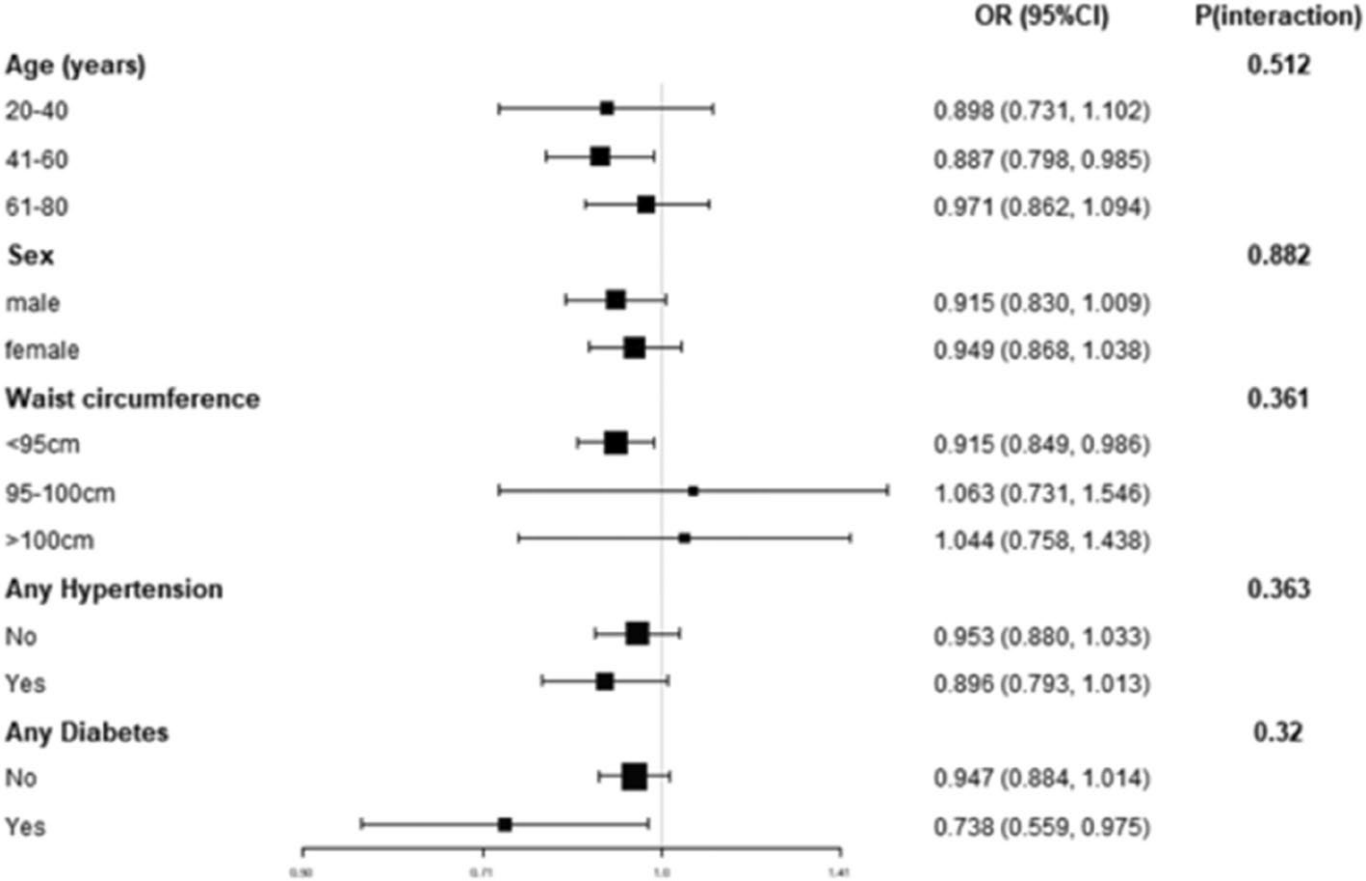
Results of subgroup analysis and interaction test (Above models adjusted for all covariates presented Table 1. In each case, the model is not adjusted for the stratification variable)

In individuals diagnosed with SDB, subgroup analysis was conducted using waist circumference as a determining factor (Fig. 2). The findings revealed a noteworthy association between the meanSpO2 and hyperlipidemia in patients with a waist circumference < 95 cm. No interactions were observed during this analysis.

### Mediation analysis

After adjusting for covariates, waist circumference was found to play a significant role in mediating the association between meanSpO2 and hyperlipidemia. Notably, the data indicated that waist circumference accounted for approximately 30.6% of the association between the meanSpO2 and hyperlipidemia (Figure S5 and Table S7).

## Discussion

To the best of our knowledge, this research constitutes the initial documentation on the utilization of type IV wearable technology for monitoring sleep patterns within a sizable community. After adjusting for covariates, the findings indicate that meanSpO2 was independently linked to dyslipidemia for all examined nocturnal blood oxygen parameters. Waist circumference may not accurately reflect noncentral body fat deposition, which tends to be more common in females [25]. BMI was included in this study’s adjusted analyses, and the results consistently supported the study findings. Additionally, when stratified by waistline, a significant association between the meanSpO2 and dyslipidemia was observed in communities without central obesity. It was intriguing to investigate which specific parameters of the lipid profile were associated with oxygen desaturation parameters [26, 27]. The present study revealed a negative correlation between minSpO2 and HDL-C levels.

The results obtained from the regression model in the present study are consistent with those reported in a previous cross-sectional study by Martínez et al. [10]. Their study focused on high-risk groups with SDB in hospitals. The analysis performed by Martínez et al. demonstrated a significant association between a decrease in meanSpO2 and dyslipidemia. This association remained significant even after adjusting for potential confounding factors such as age, sex, and BMI. However, it is important to note that the adjustments made by Martínez et al. were not comprehensive, as they did not consider other influential factors such as drinking, smoking, and exercise. Considering the close relationship between these factors and dyslipidemia, the validity of their conclusions may be questioned [28, 29]. Furthermore, another study reported a negative correlation between meanSpO2, TC, and TG levels [11]. While this particular research was distinct in terms of its target population and methodology, it had some limitations. Many prior studies have included only patients with moderate-to-severe SDB or individuals with SDB-related symptoms. In contrast, the current study sample consisted mainly of asymptomatic individuals, and the severity of SDB was not specifically classified. These broad inclusion criteria render the current study findings applicable to the general population.

The importance of subgroup analysis in scientific research cannot be overstated [30]. Unfortunately, the two previously mentioned studies did not conduct interaction testing or subgroup analysis, which hindered a comprehensive investigation of the true correlation between meanSpO2 and dyslipidemia. To address this gap, this study used age, sex, waist circumference, diabetes, and hypertension as stratifying variables for subgroup analysis. The results of the analyses indicated that there was no correlation between the meanSpO2 and dyslipidemia in the central obese population. However, in the non-central obese population, meanSpO2 was independently associated with dyslipidemia. Interestingly, among the non-obese subjects, nocturnal hypoxia resulting from SDB was linked to significantly higher triglyceride concentrations compared to those in a control group [31]. Furthermore, Li et al. made a significant discovery regarding the effects of intermittent hypoxia on lean mice. They observed that these mice exhibited a notable increase in the expression of key transcription factors that play a crucial role in the biosynthesis of triglycerides. These findings indicate a potential mechanism by which intermittent hypoxia contributes to the dysregulation of lipid metabolism [32]. Insulin effectively suppresses the production of triglycerides in the liver, and various research conducted on animals have found a potential link between intermittent hypoxia and the development of insulin resistance in lean mice [33]. In human studies, it was found that non-obese individuals with SDB experienced significant overnight increases in triglyceride levels [34]. Additionally, a small clinical study found disturbances in triglyceride metabolism in "healthy" men with severe SDB, which improved with continuous positive airway pressure (CPAP) therapy [35]. Collectively, these findings suggest that SDB-induced nocturnal hypoxia contributes to metabolic disorders involving blood lipids, particularly in non-centrally obese individuals. It is important to note that these outcomes were observed only in thin participants. Furthermore, CPAP therapy has demonstrated its efficacy in ameliorating insulin resistance to a significantly higher degree among non-obese individuals as compared to obese individuals [36].

The current study findings have significant implications in clinical settings. It is crucial for healthcare professionals to closely monitor dyslipidemia, an early indicator of atherosclerosis, in patients with SDB and nocturnal hypoxia. To assess this, the meanSpO2 can easily be extracted from a type IV sleep monitor. Of all the indexes, meanSpO2 best represents the correlation between SDB and dyslipidemia, providing valuable insights into the long-term effects of nocturnal hypoxemia. It is worth mentioning that meanSpO2 is more reliable than minSpO2 in reflecting the consequences of persistent nocturnal hypoxemia. In addition, the T90 and ODI metrics play critical roles in highlighting the impact of intermittent hypoxemia. It is essential to consider these factors, particularly in individuals with dyslipidemia and slender builds. Therefore, screening for SDB is even more important in such cases. Early detection and intervention measures must be implemented to reduce the risk of cardiovascular disease in these individuals.

According to a study conducted in the field, the prevalence of hyperlipidemia in patients with SDB is as high as 50% [37]. However, despite this high prevalence, the treatment rate for hyperlipidemia in patients with SDB is quite low, at only 12% [38]. The exact nature of the relationship between CPAP and blood lipid metabolism in patients with SDB remains unclear despite numerous clinical studies conducted on this topic and several trials investigating the impact of CPAP on blood lipid metabolism in such patients. However, these trials involved small sample sizes (< 40) and short treatment periods of no more than 6 months. Unfortunately, these trials have not shown significant changes in plasma lipids following CPAP treatment [39, 40]. A meta-analysis was conducted to further explore the impact of CPAP therapy on blood lipid levels in adult patients with SDB. This analysis included six randomized controlled trials with 348 patients and 351 controls. The results of this analysis indicated that CPAP treatment led to significant reductions in TC, TG, and HDL-C levels. However, the changes in LDL-C levels were not statistically significant [41]. Although CPAP therapy can be beneficial in treating dyslipidemia in patients with SDB, it may not be the most effective first-line treatment. Instead, lipid-lowering medications may be a more suitable option. Additionally, a 10-year follow-up study conducted by Monneret in elderly patients with SDB revealed that the proportion of high-risk dyslipidemia patients treated with statins was significantly lower in the treated group than in the untreated group [42].

## Study strengths and limitations

This study has several strengths that improved its validity and reliability. First, to ensure a diverse and representative sample, a large-scale random recruitment of community researchers was employed. Second, the potential impact of missing data on the study results was recognized and this concern was addressed using multiple interpolation methods. Third, both the linear and nonlinear regression model were used to access relationships between meanSpO2 and dyslipidemia. Interestingly, the presence of a non-linear relationship was uncovered. Fourth, a rigorous statistical adjustment technique was taken to effectively reduce any remaining confounding factors that may have influenced the lipid. Fifth, the inclusion of a subgroup analysis and interaction testing enhanced the validity and reliability of the study findings. Notably, the subgroup analysis uncovered that meanSpO2 was independently predictive of dyslipidemia in non-central obese individuals.

Although this study has many strengths, it is important to acknowledge certain limitations. First, the design of this investigation was analytical and cross-sectional, which weakened the evidence of the association between exposure and outcomes. To address this limitation, a follow-up investigation is further planned to confirm these findings. Second, the study participants were exclusively of Chinese origin, which limits the generalizability of the research outcomes to other ethnic groups.

## Conclusion

The study findings indicate that the meanSpO2 is directly associated with hyperlipidemia in community-dwelling adults with SDB. Therefore, it is crucial to consider the presence of SDB when investigating high serum triglycerides in lean males. An important conclusion drawn from this study is the importance of community general practitioners in recognizing and identifying SDB disorders at an early stage.

### Supplementary Information

**Additional file 1: Figure S1.** Type IV intelligent wearable sleep-monitoring devices and methods of use.

**Additional file 2: Figure S2**. Validation of intelligent wearable sleep monitoring devices by comparison with polysomnography.

**Additional file 3: Figure S3.** Flowchart of the study.

**Additional file 4: Figure S4.** Mediation analysis between nocturnal mean oxygen saturation and dyslipidemia in the study population using waist circumference.

**Additional file 5: Figure S5.** Correlation between nocturnal mean oxygen saturation and dyslipidemia.

**Additional file 6: Table S1.** Description of the missing data.

**Additional file 7: Table S2.** Comparative sensitivity analysis of participants with and without known lipoprotein data.

**Additional file 8: Table S3.** Results of multivariate logistic regression of post-imputation data.

**Additional file 9: Table S4.** Variance Inflation Factor collinearity screening.

**Additional file 10: Table S5.** Univariate analysis.

**Additional file 11: Table S6.** The change in the regression coefficient of the meanSpO2.

**Additional file 12: Table S7.** Mediation analysis between nocturnal mean oxygen saturation and dyslipidemia in the study population using waist circumference.

**Additional file 13: Table S8.** Baseline characteristics of the participants with and without sleep-disordered breathing.

**Additional file 14: Table S9.** Odds ratios and 95% confidence intervals for dyslipidemia in patients with sleep-disordered breathing.

**Additional file 15: Table S10.** Association of oxygen desaturation parameters with parameter of the lipid profile.

**Additional file 16: Table S11.** Association of minSpO2 with high-density lipoprotein cholesterol.

## Data Availability

Data are available from the authors upon reasonable request. The authors may be contacted at ouqiong2776@hotmail.com.

## Abbreviations

SDB: Sleep-disordered breathing
CPAP: Continuous positive airway pressure
CVD: Cardiovascular disease
TC: Total cholesterol
TG: Triglyceride
HDL-C: High-density lipoprotein cholesterol
LDL-C: Low-density lipoprotein cholesterol
ODI: Oxygen desaturation index
meanSpO2: Mean nocturnal oxygen saturation
minSpO2: Lowest recorded oxygen saturation during sleep
T90: Amount of time spent with oxygen saturation below 90%
AST: Aspartate aminotransferase
CI: Confidence interval

## References

[1] Benjafield AV, Ayas NT, Eastwood PR, Heinzer R, Ip MSM, Morrell MJ, Nunez CM, Patel SR, Penzel T, Pépin JL, et al. Estimation of the global prevalence and burden of obstructive sleep apnoea: a literature-based analysis. Lancet Respir Med. 2019;7:687–98. 31300334 10.1016/S2213-2600(19)30198-5 PMC7007763

[2] Peppard PE, Young T, Barnet JH, Palta M, Hagen EW, Hla KM. Increased prevalence of sleep-disordered breathing in adults. Am J Epidemiol. 2013;177:1006–14. 23589584 10.1093/aje/kws342 PMC3639722

[3] Redline S. Screening for obstructive sleep apnea: implications for the sleep health of the population. JAMA. 2017;317:368–70. 28118433 10.1001/jama.2016.18630

[4] Cowie MR, Linz D, Redline S, Somers VK, Simonds AK. Sleep disordered breathing and cardiovascular disease: JACC state-of-the-art review. J Am Coll Cardiol. 2021;78:608–24. 34353537 10.1016/j.jacc.2021.05.048

[5] Kendzerska T, Mollayeva T, Gershon AS, Leung RS, Hawker G, Tomlinson G. Untreated obstructive sleep apnea and the risk for serious long-term adverse outcomes: a systematic review. Sleep Med Rev. 2014;18:49–59. 23642349 10.1016/j.smrv.2013.01.003

[6] Arnaud C, Bochaton T, Pépin JL, Belaidi E. Obstructive sleep apnoea and cardiovascular consequences: Pathophysiological mechanisms. Arch Cardiovasc Dis. 2020;113:350–8. 32224049 10.1016/j.acvd.2020.01.003

[7] Peker Y, Glantz H, Eulenburg C, Wegscheider K, Herlitz J, Thunström E. Effect of positive airway pressure on cardiovascular outcomes in coronary artery disease patients with nonsleepy obstructive sleep apnea. The RICCADSA Randomized Controlled Trial. Am J Respir Crit Care Med. 2016;194:613–620. 10.1164/rccm.201601-0088OC 26914592

[8] Sánchez-de-la-Torre M, Sánchez-de-la-Torre A, Bertran S, Abad J, Duran-Cantolla J, Cabriada V, Mediano O, Masdeu MJ, Alonso ML, Masa JF, et al. Effect of obstructive sleep apnoea and its treatment with continuous positive airway pressure on the prevalence of cardiovascular events in patients with acute coronary syndrome (ISAACC study): a randomised controlled trial. Lancet Respir Med. 2020;8:359–67. 31839558 10.1016/S2213-2600(19)30271-1

[9] Sun Z, Shen W. Effect of intermittent hypoxia on lipid metabolism in liver cells and the underlying mechanism. Zhonghua Gan Zang Bing Za Zhi. 2014;22:369–73. 25180873 10.3760/cma.j.issn.1007-3418.2014.05.010

[10] Martínez-Cerón E, Casitas R, Galera R, Sánchez-Sánchez B, Zamarrón E, Garcia-Sanchez A, Jaureguizar A, Cubillos-Zapata C, Garcia-Rio F. Contribution of sleep characteristics to the association between obstructive sleep apnea and dyslipidemia. Sleep Med. 2021;84:63–72. 34111805 10.1016/j.sleep.2021.05.012

[11] Silva LOE, Guimarães TM, Luz GP, Coelho G, Badke L, Almeida IR, Millani-Carneiro A, Tufik S, Bittencourt L, Togeiro SM. Metabolic profile in patients with mild obstructive sleep apnea. Metab Syndr Relat Disord. 2018;16:6–12. 29148894 10.1089/met.2017.0075

[12] Trzepizur W, Le Vaillant M, Meslier N, Pigeanne T, Masson P, Humeau MP, Bizieux-Thaminy A, Goupil F, Chollet S, Ducluzeau PH, Gagnadoux F. Independent association between nocturnal intermittent hypoxemia and metabolic dyslipidemia. Chest. 2013;143:1584–9. 23699812 10.1378/chest.12-1652

[13] Guscoth LB, Appleton SL, Martin SA, Adams RJ, Melaku YA, Wittert GA. The association of obstructive sleep apnea and nocturnal hypoxemia with lipid profiles in a population-based study of community-dwelling Australian men. Nat Sci Sleep. 2021;13:1771–82. 34675725 10.2147/NSS.S327478 PMC8517637

[14] Kuvat N, Tanriverdi H, Armutcu F. The relationship between obstructive sleep apnea syndrome and obesity: a new perspective on the pathogenesis in terms of organ crosstalk. Clin Respir J. 2020;14:595–604. 32112481 10.1111/crj.13175

[15] Bonsignore MR. Obesity and obstructive sleep apnea. Handb Exp Pharmacol. 2022;274:181–201. 34697666 10.1007/164_2021_558

[16] He H, Pan L, Pa L, Cui Z, Ren X, Wang D, Liu F, Wang X, Du J, Wang H, et al. Data resource profile: The China National Health Survey (CNHS). Int J Epidemiol. 2018;47:1734–1735f. 30124853 10.1093/ije/dyy151

[17] Xu Y, Ou Q, Cheng Y, Lao M, Pei G. Comparative study of a wearable intelligent sleep monitor and polysomnography monitor for the diagnosis of obstructive sleep apnea. Sleep Breath. 2022;27:205–12. 10.1007/s11325-022-02599-x PMC9992231 35347656

[18] Joint Committee for Developing Chinese guidelines on Prevention and Treatment of Dyslipidemia in Adults. Chinese guidelines on prevention and treatment of dyslipidemia in adults. Zhonghua Xin Xue Guan Bing Za Zhi. 2007;35:390–419. 17711682

[19] Tsutsui JM, Xie F, Cloutier D, Kalvaitis S, Elhendy A, Porter TR. Real-time dobutamine stress myocardial perfusion echocardiography predicts outcome in the elderly. Eur Heart J. 2008;29:377–85. 17989076 10.1093/eurheartj/ehm445

[20] Sui X, Hooker SP, Lee IM, Church TS, Colabianchi N, Lee CD, Blair SN. A prospective study of cardiorespiratory fitness and risk of type 2 diabetes in women. Diabetes Care. 2008;31:550–5. 18070999 10.2337/dc07-1870 PMC3410433

[21] Kaddourah A, Basu RK, Bagshaw SM, Goldstein SL. Epidemiology of acute kidney injury in critically Ill children and young adults. N Engl J Med. 2017;376:11–20. 27959707 10.1056/NEJMoa1611391 PMC5322803

[22] Park SY, Freedman ND, Haiman CA, Le Marchand L, Wilkens LR, Setiawan VW. Association of coffee consumption with total and cause-specific mortality among nonwhite populations. Ann Intern Med. 2017;167:228–35. 28693036 10.7326/M16-2472 PMC7494322

[23] Bernhardt PW. Model validation and influence diagnostics for regression models with missing covariates. Stat Med. 2018;37:1325–42. 29318652 10.1002/sim.7584

[24] Jaddoe VW, de Jonge LL, Hofman A, Franco OH, Steegers EA, Gaillard R. First trimester fetal growth restriction and cardiovascular risk factors in school age children: population based cohort study. BMJ. 2014;348: g14. 24458585 10.1136/bmj.g14 PMC3901421

[25] Bikov A, Frent SM, Meszaros M, Kunos L, Mathioudakis AG, Negru AG, Gaita L, Mihaicuta S. Triglyceride-glucose index in non-diabetic, non-obese patients with obstructive sleep apnoea. J Clin Med. 2021;10:1932. 10.3390/jcm10091932 PMC8125770 33947164

[26] Bikov A, Meszaros M, Kunos L, Negru AG, Frent SM, Mihaicuta S. Atherogenic index of plasma in obstructive sleep apnoea. J Clin Med. 2021;10:417. 10.3390/jcm10030417 PMC7865393 33499142

[27] Bikov A, Frent S, Reisz D, Negru A, Gaita L, Breban Schwarzkopf D, Mihaicuta S. Comparison of composite lipid indices in patients with obstructive sleep apnoea. Nat Sci Sleep. 2022;14:1333–40. 35923809 10.2147/NSS.S361318 PMC9342428

[28] Mann S, Beedie C, Jimenez A. Differential effects of aerobic exercise, resistance training and combined exercise modalities on cholesterol and the lipid profile: review, synthesis and recommendations. Sports Med. 2014;44:211–21. 24174305 10.1007/s40279-013-0110-5 PMC3906547

[29] Li XX, Zhao Y, Huang LX, Xu HX, Liu XY, Yang JJ, Zhang PJ, Zhang YH. Effects of smoking and alcohol consumption on lipid profile in male adults in northwest rural China. Public Health. 2018;157:7–13. 29459348 10.1016/j.puhe.2018.01.003

[30] Vandenbroucke JP, von Elm E, Altman DG, Gøtzsche PC, Mulrow CD, Pocock SJ, Poole C, Schlesselman JJ, Egger M. Strengthening the reporting of observational studies in epidemiology (STROBE): explanation and elaboration. PLoS Med. 2007;4: e297. 17941715 10.1371/journal.pmed.0040297 PMC2020496

[31] Lin QC, Zhang XB, Chen GP, Huang DY, Din HB, Tang AZ. Obstructive sleep apnea syndrome is associated with some components of metabolic syndrome in nonobese adults. Sleep Breath. 2012;16:571–8. 21681412 10.1007/s11325-011-0544-7

[32] Li J, Thorne LN, Punjabi NM, Sun CK, Schwartz AR, Smith PL, Marino RL, Rodriguez A, Hubbard WC, O’Donnell CP, Polotsky VY. Intermittent hypoxia induces hyperlipidemia in lean mice. Circ Res. 2005;97:698–706. 16123334 10.1161/01.RES.0000183879.60089.a9

[33] Iiyori N, Alonso LC, Li J, Sanders MH, Garcia-Ocana A, O’Doherty RM, Polotsky VY, O’Donnell CP. Intermittent hypoxia causes insulin resistance in lean mice independent of autonomic activity. Am J Respir Crit Care Med. 2007;175:851–7. 17272786 10.1164/rccm.200610-1527OC PMC1899294

[34] Koenig AM, Koehler U, Hildebrandt O, Schwarzbach H, Hannemann L, Boneberg R, Heverhagen JT, Mahnken AH, Keller M, Kann PH, et al. The effect of obstructive sleep apnea and continuous positive airway pressure therapy on skeletal muscle lipid content in obese and nonobese men. J Endocr Soc. 2021;5:bvab082. 10.1210/jendso/bvab082 PMC8274947 34268461

[35] Drager LF, Tavoni TM, Silva VM, Santos RD, Pedrosa RP, Bortolotto LA, Vinagre CG, Polotsky VY, Lorenzi-Filho G, Maranhao RC. Obstructive sleep apnea and effects of continuous positive airway pressure on triglyceride-rich lipoprotein metabolism. J Lipid Res. 2018;59:1027–33. 29628442 10.1194/jlr.M083436 PMC5983397

[36] Harsch IA, Schahin SP, Radespiel-Tröger M, Weintz O, Jahreiss H, Fuchs FS, Wiest GH, Hahn EG, Lohmann T, Konturek PC, Ficker JH. Continuous positive airway pressure treatment rapidly improves insulin sensitivity in patients with obstructive sleep apnea syndrome. Am J Respir Crit Care Med. 2004;169:156–62. 14512265 10.1164/rccm.200302-206OC

[37] Wang L, Ou Q, Shan G, Lao M, Xu Y, Pei G. Distinct phenotypic clusters of sleep-disordered breathing and their association with medical care-seeking behaviour and sleep habits: the Guangdong Sleep Health Study. J Sleep Res. 2022;32:e13762. 10.1111/jsr.13762 36325765

[38] Gunduz C, Basoglu OK, Hedner J, Bonsignore MR, Hein H, Staats R, Bouloukaki I, Roisman G, Pataka A, Sliwinski P, et al. Hyperlipidaemia prevalence and cholesterol control in obstructive sleep apnoea: data from the European sleep apnea database (ESADA). J Intern Med. 2019;286:676–88. 31260567 10.1111/joim.12952

[39] Drager LF, Bortolotto LA, Figueiredo AC, Krieger EM, Lorenzi GF. Effects of continuous positive airway pressure on early signs of atherosclerosis in obstructive sleep apnea. Am J Respir Crit Care Med. 2007;176:706–12. 17556718 10.1164/rccm.200703-500OC

[40] Comondore VR, Cheema R, Fox J, Butt A, John Mancini GB, Fleetham JA, Ryan CF, Chan S, Ayas NT. The impact of CPAP on cardiovascular biomarkers in minimally symptomatic patients with obstructive sleep apnea: a pilot feasibility randomized crossover trial. Lung. 2009;187:17–22. 18795367 10.1007/s00408-008-9115-5

[41] Lin MT, Lin HH, Lee PL, Weng PH, Lee CC, Lai TC, Liu W, Chen CL. Beneficial effect of continuous positive airway pressure on lipid profiles in obstructive sleep apnea: a meta-analysis. Sleep Breath. 2015;19:809–17. 25450153 10.1007/s11325-014-1082-x PMC4559086

[42] Monneret D, Barthélémy JC, Hupin D, Maudoux D, Celle S, Sforza E, Roche F. Serum lipid profile, sleep-disordered breathing and blood pressure in the elderly: a 10-year follow-up of the PROOF-SYNAPSE cohort. Sleep Med. 2017;39:14–22. 29157582 10.1016/j.sleep.2017.07.028

