# Correction: The association of nocturnal hypoxemia with dyslipidemia in sleep‑disordered breathing population of Chinese community: a cross‑sectional study

**DOI:** 10.1186/s12944-024-02208-8

**Published:** 2024-07-31

**Authors:** Tong Feng, Guangliang Shan, Huijing He, Guo Pei, Jiaoying Tan, Bing Lu, Qiong Ou

**Affiliations:** 1 The Second School of Clinical Medicine, Southern Medical University, Guangzhou, China; 2 Sleep Center, Department of Respiratory and Critical Care MedicineGuangdong Provincial People’s Hospital (Guangdong Academy of Medical Sciences)Yuexiu District, Southern Medical University, No.106 Zhongshan Road, Guangzhou City, Guangdong Province, China; 3 Department of Epidemiology & Biostatistics, Institute of Basic Medical Sciences, Chinese Academy of Medical Sciences/School of Basic Medicine, Peking Union Medical College, Beijing, People’s Republic of China

**Correction: Lipids Health Dis 22, 159 (2023)**

10.1186/s12944-023-01919-8

Following publication of the original article [1], the authors reported an error found in Baseline characteristics of participants section and in Table 1.

The updated Baseline characteristics of participants is given below and the changes have been highlighted in **bold typeface**.

Baseline characteristics of participants:

The flow chart representing the process of research registration can be found in Figure S3, whereas Table 1 highlights the distribution of clinical characteristics among 1052 individuals with SDB residing in a community. The participants’ age varied from 20 to 90 years, with an average age of 56.15 ± 13.11 years. Among the total sample, 55.7% were identified as male. It is worth mentioning that individuals diagnosed with hyperlipidemia were comparatively **older** and exhibited lower MeanSpO2 values when compared to those individuals without hyperlipidemia. No significant variations were observed in the distribution of ODI, minSpO2, or T90 between the two groups.

In Table 1, the labels “Without Dyslipidaemia (n = 560)” and “With Dyslipidaemia (n = 492)” are incorrect and should be reversed. The correct labels should be:

 Without Dyslipidaemia (*n* = 492)

 With Dyslipidaemia (*n* = 560

The correct Table 1 is presented below:

**Table 1:** Baseline characteristics of participants

Characteristics	All Participants (*n* = 1052)	Without Dyslipidaemia (*n* = 492)	With Dyslipidaemia (*n* = 560)	*P*-value
Age (years)	56.15 ± 13.11	54.99 ± 13.97	57.47 ± 11.95	0.002
Sex (%)				0.004
Female	466 (44.30%)	225 (40.18%)	241 (48.98%)	
Male	586 (55.70%)	335 (59.82%)	251 (51.02%)	
Martial Status (%)				0.042
Single	43 (4.09%)	31 (5.54%)	12 (2.44%)	
Married	929 (88.31%)	490 (87.50%)	439 (89.23%)	
Divorce	12 (1.14%)	8 (1.43%)	4 (0.81%)	
Widowed	67 (6.37%)	30 (5.36%)	37 (7.52%)	
Education (%)				0.171
Less than high school	499 (47.43%)	253 (45.18%)	246 (50.00%)	
High school	292 (27.76%)	156 (27.86%)	136 (27.64%)	
More than high school	261 (24.81%)	151 (26.96%)	110 (22.36%)	
Waist circumference(%)				0.124
< 95 cm	839 (79.75%)	461 (82.32%)	378 (76.83%)	
95–100 cm	98 (9.32%)	43 (7.68%)	55 (11.18%)	
> 100 cm	79 (7.51%)	40 (7.14%)	39 (7.93%)	
Physical exercise (%)				0.524
5–7 days per week	588 (55.89%)	316 (56.43%)	272 (55.28%)	
3–4 days per week	101 (9.60%)	57 (10.18%)	44 (8.94%)	
1–2 days per week	108 (10.27%)	61 (10.89%)	47 (9.55%)	
≤ 3 days per month	77 (7.32%)	35 (6.25%)	42 (8.54%)	
Never exercising	177 (16.83%)	91 (16.25%)	86 (17.48%)	
Cigarette smoking(%)				< 0.001
No	805 (76.52%)	449 (80.18%)	356 (72.36%)	
Former	66 (6.27%)	38 (6.79%)	28 (5.69%)	
Current	181 (17.21%)	73 (13.04%)	108 (21.95%)	
Alcohol use (%)				0.102
No	818 (77.76%)	447 (79.82%)	371 (75.41%)	
Former	23 (2.19%)	14 (2.50%)	9 (1.83%)	
Current	211 (20.06%)	99 (17.68%)	112 (22.76%)	
Diabetes(%)				0.141
No	905 (86.03%)	490 (87.50%)	415 (84.35%)	
Yes	147 (13.97%)	70 (12.50%)	77 (15.65%)	
Hypertension(%)				0.003
No	640 (60.84%)	364 (65.00%)	276 (56.10%)	
Yes	412 (39.16%)	196 (35.00%)	216 (43.90%)	
MeanSpO2(%)	95.42 ± 2.02	95.59 ± 1.96	95.23 ± 2.06	0.004
MinSpO2(%)	81.85 ± 4.96	81.95 ± 4.80	81.75 ± 5.14	0.514
T90(%)	5.06 ± 10.32	4.98 ± 9.98	5.16 ± 10.71	0.789
T90(s)	1060.07 ± 2053.64	1039.48 ± 1979.30	1083.51 ± 2136.89	0.729
ODI( events/h)	13.80 ± 7.59	13.46 ± 7.16	14.18 ± 8.04	0.122
Total cholesterol (mmol/L)	5.65 ± 1.07	5.21 ± 0.67	6.16 ± 1.20	< 0.001
HDL-C (mmol/L)	1.34 ± 0.38	1.45 ± 0.33	1.21 ± 0.40	< 0.001
LDL-C (mmol/L)	3.30 ± 0.87	3.01 ± 0.57	3.64 ± 1.01	< 0.001
Triglycerides (mmol/L)	1.66 ± 1.25	1.14 ± 0.41	2.25 ± 1.58	< 0.001
AST (U/L)	24.78 ± 10.72	24.19 ± 11.69	25.45 ± 9.47	0.057
Fasting blood glucose(mmol/L)	6.00 ± 1.45	5.88 ± 1.25	6.13 ± 1.65	0.006
Creatinine(umol/l)	74.66 ± 22.75	72.93 ± 23.25	76.63 ± 22.02	0.008
