# Supplementary material for: The association of nocturnal hypoxemia with dyslipidemia in sleep-disordered breathing population of Chinese community: a cross-sectional study
Source: Lipids Health Dis. 2023 Sep 26;22:159. doi: 10.1186/s12944-023-01919-8 (PMC10521560; doi:10.1186/s12944-023-01919-8)
Supplement: Supplementary file 1 — Additional file 1: Figure S1. Type IV intelligent wearable sleep-monitoring devices and methods of use. [file 12944_2023_1919_MOESM1_ESM.doc]

**Figure S1. Type IV intelligent wearable sleep monitoring devices and method of use**

(A)

**
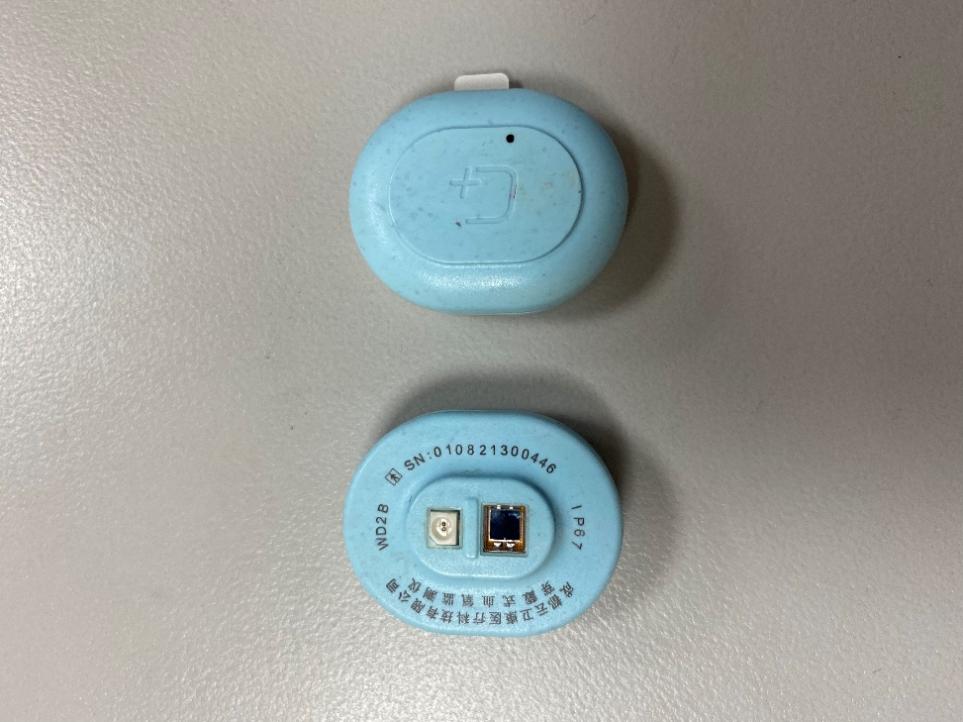
**

(B)

**
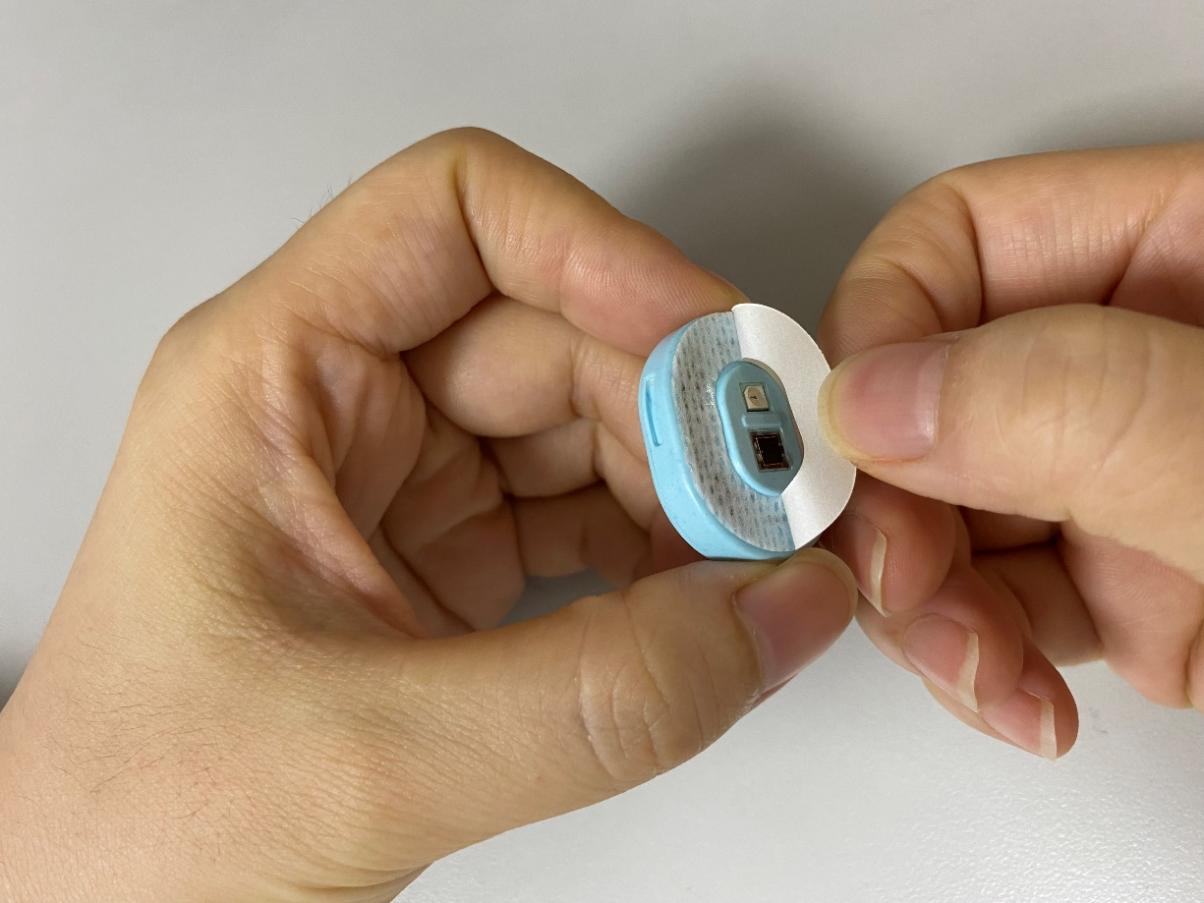
**

(C)

**
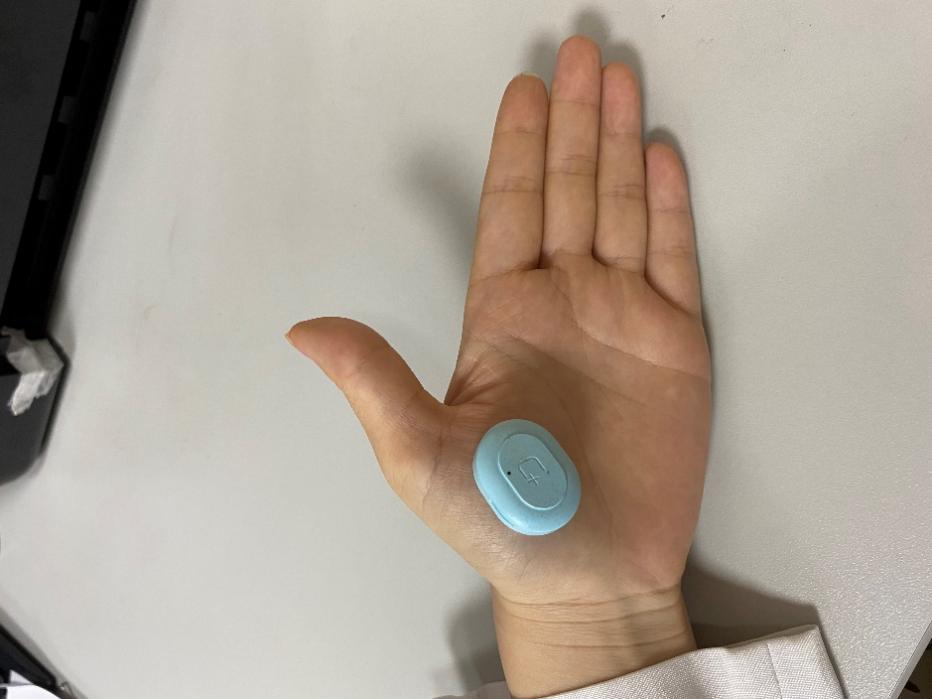
**

(A) Front and back photos of intelligent wearable sleep monitoring devices

(B) Double-sided adhesive is attached to the side with the photoelectric reflex sensor

(C) The device is tightly adhered to the position of palmar thenar major muscles with double-sided adhesive
