# Supplementary material for: The association of nocturnal hypoxemia with dyslipidemia in sleep-disordered breathing population of Chinese community: a cross-sectional study
Source: Lipids Health Dis. 2023 Sep 26;22:159. doi: 10.1186/s12944-023-01919-8 (PMC10521560; doi:10.1186/s12944-023-01919-8)
Supplement: Supplementary file 2 — Additional file 2: Figure S2. Validation of intelligent wearable sleep monitoring devices by comparison with polysomnography. [file 12944_2023_1919_MOESM2_ESM.doc]

**Figure S2. Validation of intelligent wearable sleep monitoring devices by comparison against polysomnography**

(A)

(B)


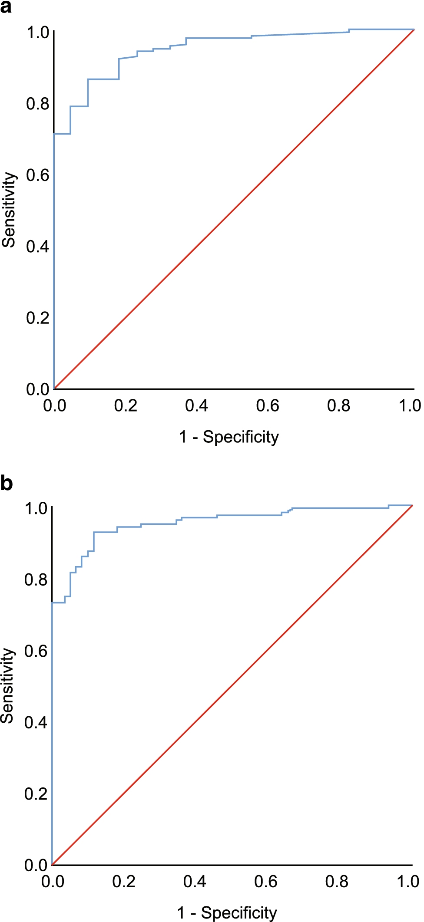

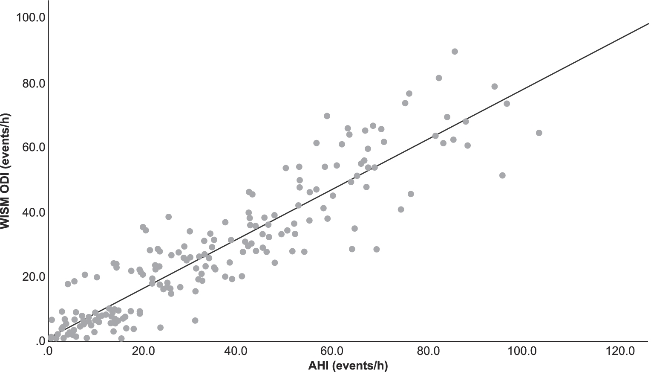


**(A) ROC curve of SDB diagnosed by WISM at AHI threshold ≥ 5 times/hour as defined by PSG. (B) AHI and WISM ODI conformance analysis.**

Note: Figures (A) and (B) are from the previously published study of our research team; Adapted with permission from Xu Y, Ou Q, Cheng Y, et al. Comparative study of a wearable intelligent sleep monitor and polysomnography monitor for the diagnosis of obstructive sleep apnea. *Sleep Breath*, 2022.^1^

Our research team validated the wearable intelligent sleep monitoring device (WISM) by comparison against polysomnography (PSG) at the Sleep Center of Guangdong Provincial People's Hospital from July 2020 to March 2021. In total, 196 participants completed both PSG and WISM monitoring at the same time. When the ODI by WISM reaches 7.0 events/h, the optimal cut-off value, it has a sensitivity, specificity, and the area under the ROC curve of 86%, 91%, and 0.95, respectively, to predict SDB defined by an apnea-hypopnea index (AHI) ≥5 events/h (Figure (A)). In addition, the ODI had a strong correlation with the AHI from the PSG (R ^2^ =0.843, P < 0.001, Figure (B)).

SDB= sleep-disordered breathing; ROC= receiver-operating characteristic; WISM= wearable intelligent sleep monitor; AHI apnea–hypopnea index; ODI= oxygen desaturation index; PSG= polysomnography

References

[1] Xu Y, Ou Q, Cheng Y, et al. Comparative study of a wearable intelligent sleep monitor and polysomnography monitor for the diagnosis of obstructive sleep apnea [J]. Sleep Breath, 2022. DOI: 10.1007/s11325-022-02599-x.
