# Supplementary figures and images for: The association of nocturnal hypoxemia with dyslipidemia in sleep-disordered breathing population of Chinese community: a cross-sectional study
Source: Lipids Health Dis. 2023 Sep 26;22:159. doi: 10.1186/s12944-023-01919-8 (PMC10521560; doi:10.1186/s12944-023-01919-8)

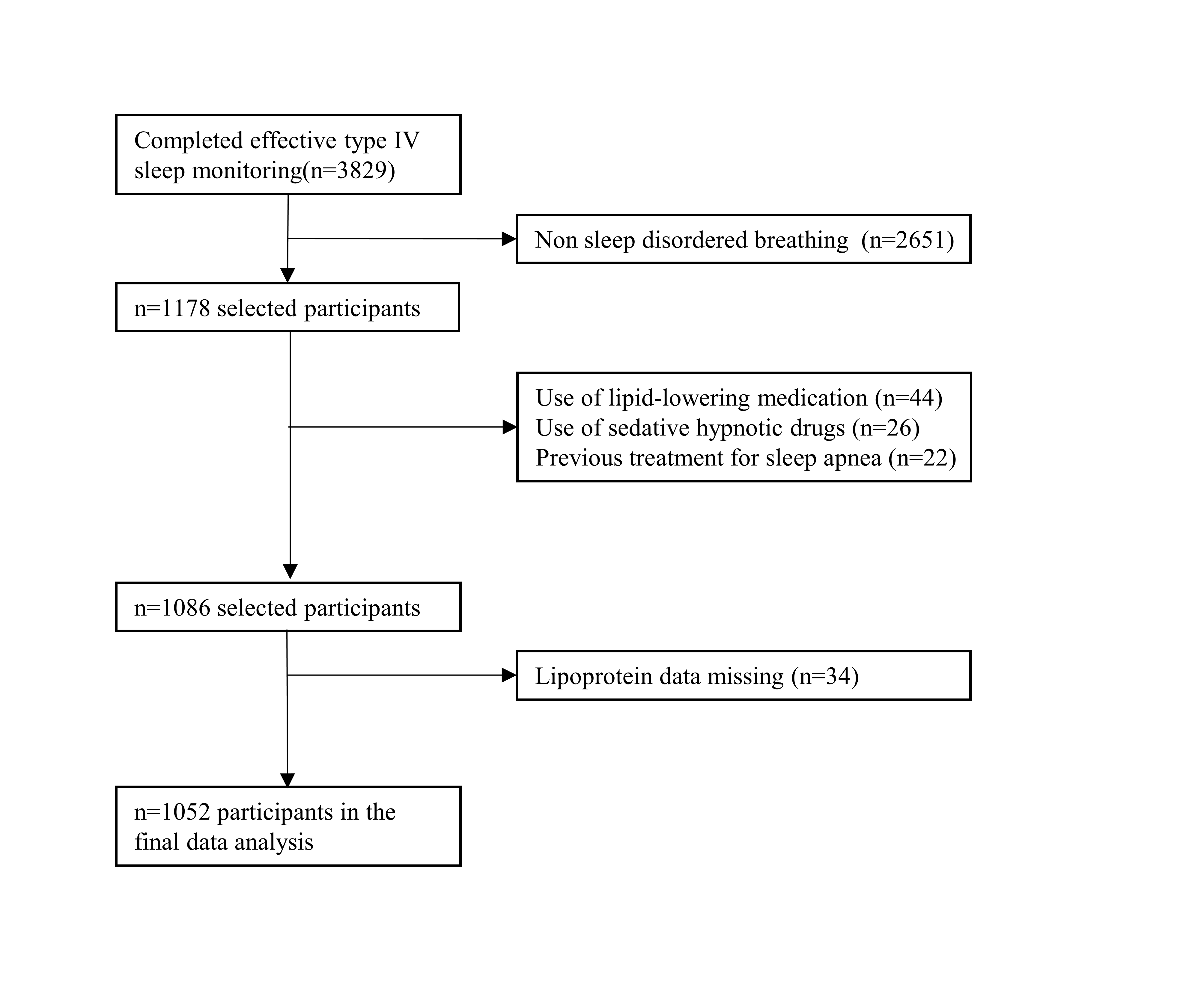

Supplement: Supplementary file 3 — Additional file 3: Figure S3. Flowchart of the study. [file 12944_2023_1919_MOESM3_ESM.tif]

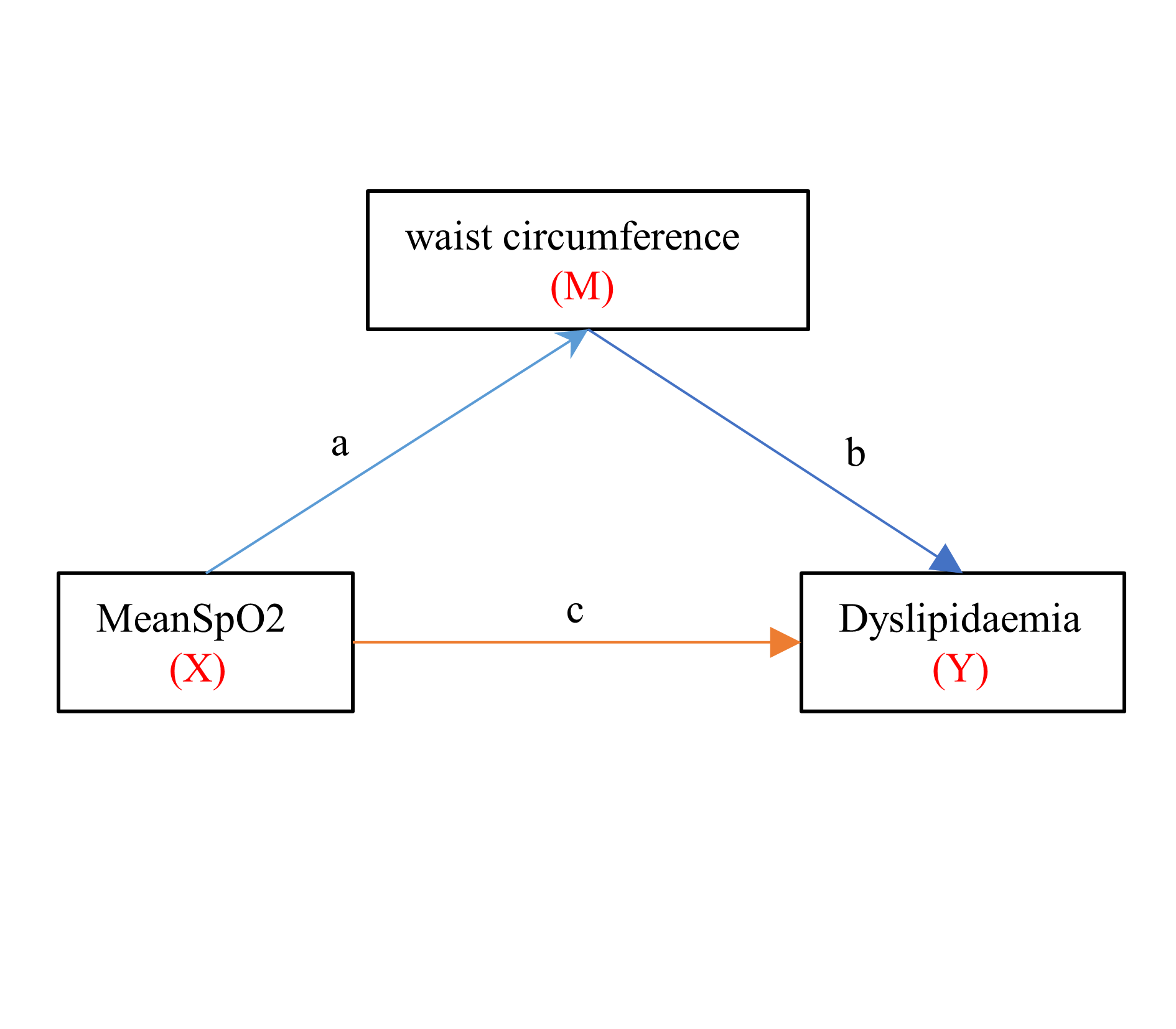

Supplement: Supplementary file 4 — Additional file 4: Figure S4. Mediation analysis between nocturnal mean oxygen saturation and dyslipidemia in the study population using waist circumference. [file 12944_2023_1919_MOESM4_ESM.tif]

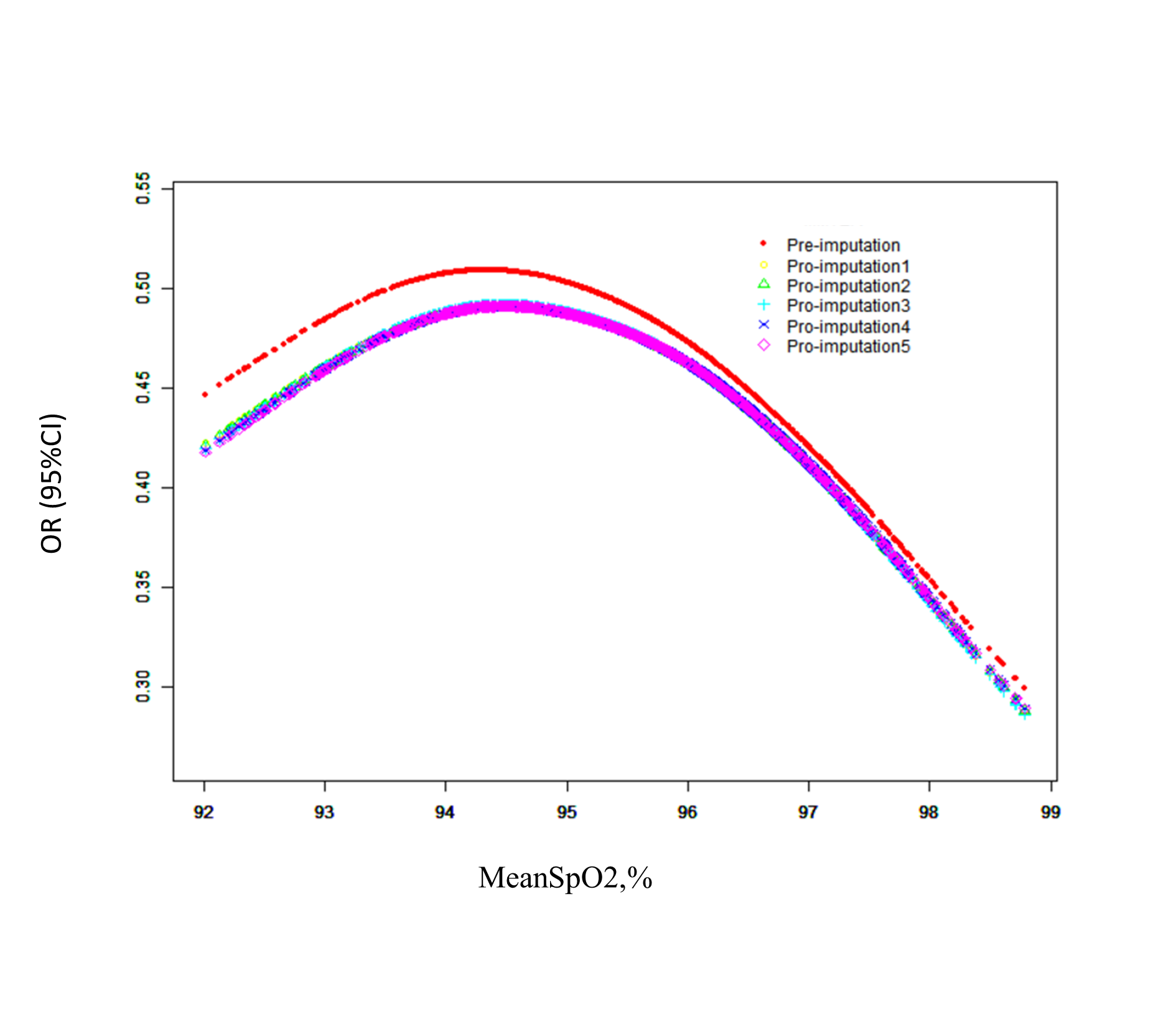

Supplement: Supplementary file 5 — Additional file 5: Figure S5. Correlation between nocturnal mean oxygen saturation and dyslipidemia. [file 12944_2023_1919_MOESM5_ESM.tif]
