# Supplementary material for: The association of nocturnal hypoxemia with dyslipidemia in sleep-disordered breathing population of Chinese community: a cross-sectional study
Source: Lipids Health Dis. 2023 Sep 26;22:159. doi: 10.1186/s12944-023-01919-8 (PMC10521560; doi:10.1186/s12944-023-01919-8)
Supplement: Supplementary file 6 — Additional file 6: Table S1. Description of the missing data. [file 12944_2023_1919_MOESM6_ESM.doc]

**Table S1. The description of missing data**

| Variables | Non-missing | missing |
| --- | --- | --- |
| Waist circumference | 1044 | 42 |
| Sex | 1086 | 0 |
| Age | 1085 | 1 |
| Total cholesterol | 1052 | 34 |
| HDL-C | 1052 | 34 |
| LDL-C | 1052 | 34 |
| Triglycerides | 1052 | 34 |
| ODI | 1086 | 0 |
| MeanSpO2 | 1086 | 0 |
| MinSpO2 | 1086 | 0 |
| T90% | 1086 | 0 |
| T90(s) | 1086 | 0 |
| Martial Status | 1052 | 34 |
| Education | 1053 | 33 |
| Physical exercise | 1052 | 34 |
| Cigarette smoking | 1053 | 33 |
| Alcohol use | 1053 | 33 |
| Diabetes | 1053 | 33 |
| Hypertension | 1053 | 33 |
| Ast | 1052 | 34 |
| Glu | 1052 | 34 |
| Crea | 1052 | 34 |
