# Supplementary material for: The association of nocturnal hypoxemia with dyslipidemia in sleep-disordered breathing population of Chinese community: a cross-sectional study
Source: Lipids Health Dis. 2023 Sep 26;22:159. doi: 10.1186/s12944-023-01919-8 (PMC10521560; doi:10.1186/s12944-023-01919-8)
Supplement: Supplementary file 7 — Additional file 7: Table S2. Comparative sensitivity analysis of participants with and without known lipoprotein data. [file 12944_2023_1919_MOESM7_ESM.doc]

**Table S2. Sensitivity comparative analysis between participants with vs without known lipoproteins data**

|  | Participants with known lipoproteins data  N=1052 | Participants with missing lipoproteins data  N=34 | P-value |
| --- | --- | --- | --- |
| Age (years) | 56.146 ± 13.112 | 57.788 ± 12.113 | 0.478 |
| Waist circumference(cm) | 86.138 ± 9.775 | 87.432 ± 10.154 | 0.490 |
| MeanSpO2 | 95.422 ± 2.018 | 95.490 ± 2.006 | 0.846 |
| MinSpO2 | 81.855 ± 4.959 | 84.294 ± 3.623 | 0.005 |
| T90% | 17.668 ± 34.227 | 14.205 ± 42.206 | 0.565 |
| T90(s) | 1060.073 ± 2053.639 | 852.294 ± 2532.341 | 0.565 |
| ODI( events/h) | 13.799 ± 7.589 | 12.168 ± 5.067 | 0.214 |
| AST (U/L) | 24.777 ± 10.723 | - |  |
| Fasting blood glucose(mmol/L) | 5.995 ± 1.451 | - |  |
| Creatinine(umol/l) | 74.661 ± 22.748 | - |  |
| Sex (%) |  |  | 0.718 |
| Female | 466 (44.297%) | 14 (41.176%) |  |
| Male | 586 (55.703%) | 20 (58.824%) |  |
| Martial Status (%) |  |  | 0.988 |
| Single | 43 (4.091%) | 0 (0.000%) |  |
| Married | 929 (88.392%) | 1 (100.000%) |  |
| Divorce | 12 (1.142%) | 0 (0.000%) |  |
| Widowed | 67 (6.375%) | 0 (0.000%) |  |
| Education (%) |  |  | 0.575 |
| Less than high school | 499 (47.433%) | 1 (100.000%) |  |
| High school | 292 (27.757%) | 0 (0.000%) |  |
| More than high school | 261 (24.810%) | 0 (0.000%) |  |
| Physical exercise (%) |  |  | 0.940 |
| 5-7 days per week | 588 (55.947%) | 1 (100.000%) |  |
| 3-4 days per week | 101 (9.610%) | 0 (0.000%) |  |
| 1-2 days per week | 108 (10.276%) | 0 (0.000%) |  |
| ≤ 3 days per month | 77 (7.326%) | 0 (0.000%) |  |
| never exercising | 177 (16.841%) | 0 (0.000%) |  |
| Cigarette smoking(%) |  |  | 0.858 |
| No | 805 (76.521%) | 1 (100.000%) |  |
| Former | 66 (6.274%) | 0 (0.000%) |  |
| Current | 181 (17.205%) | 0 (0.000%) |  |
| Alcohol use (%) |  |  | 0.867 |
| No | 818 (77.757%) | 1 (100.000%) |  |
| Former | 23 (2.186%) | 0 (0.000%) |  |
| Current | 211 (20.057%) | 0 (0.000%) |  |
| Diabetes(%) |  |  | 0.687 |
| No | 905 (86.027%) | 1 (100.000%) |  |
| Yes | 147 (13.973%) | 0 (0.000%) |  |
| Hypertension(%) |  |  | 0.422 |
| No | 640 (60.837%) | 1 (100.000%) |  |
| Yes | 412 (39.163%) | 0 (0.000%) |  |

Nearly all variables were similar in patients with available data on lipoproteins and the 34 participants with missing data on lipoproteins.
