# Supplementary material for: The association of nocturnal hypoxemia with dyslipidemia in sleep-disordered breathing population of Chinese community: a cross-sectional study
Source: Lipids Health Dis. 2023 Sep 26;22:159. doi: 10.1186/s12944-023-01919-8 (PMC10521560; doi:10.1186/s12944-023-01919-8)
Supplement: Supplementary file 8 — Additional file 8: Table S3. Results of multivariate logistic regression of post-imputation data. [file 12944_2023_1919_MOESM8_ESM.doc]

**Table S3.Results of multivariate logistic regression among post-imputation data**

|  | Pro-imputation1  OR,(95%CI) | Pro-imputation2  OR,(95%CI) | Pro-imputation3  OR,(95%CI) | Pro-imputation4  OR,(95%CI) | Pro-imputation5  OR,(95%CI) | Pooled by rubin’s rule  OR,(95%CI) |
| --- | --- | --- | --- | --- | --- | --- |
| Model 1 | 0.915 (0.860, 0.974) | 0.915 (0.860, 0.974) | 0.915 (0.860, 0.974) | 0.915 (0.860, 0.974) | 0.915 (0.860, 0.974) | 0.915 (0.860, 0.974) |
| Model 2 | 0.930 (0.874, 0.989) | 0.930 (0.874, 0.989) | 0.930 (0.874, 0.990) | 0.930 (0.874, 0.990) | 0.930 (0.874, 0.990) | 0.930 (0.874, 0.990) |
| Model 3 | 0.930 (0.873, 0.991) | 0.932 (0.875, 0.993) | 0.931 (0.874, 0.992) | 0.929 (0.873, 0.990) | 0.929 (0.872, 0.990) | 0.930 (0.873, 0.991) |

Notes: Model 1: no covariates were adjusted

Model 2: only sociodemographic variables were adjusted (age, sex, education level, marital status)

Model 3: all covariates presented in Table 1 were adjusted
