# Supplementary material for: The association of nocturnal hypoxemia with dyslipidemia in sleep-disordered breathing population of Chinese community: a cross-sectional study
Source: Lipids Health Dis. 2023 Sep 26;22:159. doi: 10.1186/s12944-023-01919-8 (PMC10521560; doi:10.1186/s12944-023-01919-8)
Supplement: Supplementary file 9 — Additional file 9: Table S4. Variance Inflation Factor collinearity screening. [file 12944_2023_1919_MOESM9_ESM.doc]

**Covariant check and screening**
**Table S4.Variance Inflation Factor collinearity screening**

|  | variance inflation factor |
| --- | --- |
| MEANSPO2 | 1 |
| SEX | 2.3 |
| AGE | 1.7 |
| EDU_CATEGORY3 | 1.4 |
| SMOKE | 1.7 |
| DRINK | 1.3 |
| AST | 1 |
| CREA | 1.5 |
| DIABETES | 2.2 |
| GLU | 2.2 |
| HYPERTENSION | 1.2 |
| MARITAL.1 | 1.2 |
| EXERCISE.1 | 1.1 |
| ECONOMIC.1 | 1.1 |
| WC.3 | 1.1 |
