# Supplementary material for: The association of nocturnal hypoxemia with dyslipidemia in sleep-disordered breathing population of Chinese community: a cross-sectional study
Source: Lipids Health Dis. 2023 Sep 26;22:159. doi: 10.1186/s12944-023-01919-8 (PMC10521560; doi:10.1186/s12944-023-01919-8)
Supplement: Supplementary file 10 — Additional file 10: Table S5. Univariate analysis. [file 12944_2023_1919_MOESM10_ESM.doc]

**Table S5.Univariate analysis**

| Covariates | N | term | beta | Se. | exp(beta) | 95%CI Low | 95%CI Upp | P.value |
| --- | --- | --- | --- | --- | --- | --- | --- | --- |
| SEX | 1052 | factor(SEX)2 | -0.3574 | 0.1248 | 0.6995 | 0.5478 | 0.8933 | 0.0042 |
| AGE | 1052 | AGE | 0.0146 | 0.0048 | 1.0147 | 1.0052 | 1.0243 | 0.0023 |
| EDU_CATEGORY3 | 1052 | factor(EDU_CATEGORY3)2 | -0.1091 | 0.1476 | 0.8966 | 0.6714 | 1.1974 | 0.4596 |
|  |  | factor(EDU_CATEGORY3)3 | -0.2887 | 0.1540 | 0.7492 | 0.5540 | 1.0133 | 0.0609 |
| SMOKE | 1052 | factor(SMOKE)1 | -0.0733 | 0.2590 | 0.9293 | 0.5594 | 1.5439 | 0.7772 |
|  |  | factor(SMOKE)2 | 0.6238 | 0.1673 | 1.8659 | 1.3442 | 2.5901 | 0.0002 |
| DRINK | 1052 | factor(DRINK)1 | -0.2555 | 0.4329 | 0.7745 | 0.3315 | 1.8096 | 0.5551 |
|  |  | factor(DRINK)2 | 0.3097 | 0.1548 | 1.3631 | 1.0064 | 1.8462 | 0.0454 |
| AST | 1052 | AST | 0.0116 | 0.0063 | 1.0117 | 0.9993 | 1.0242 | 0.0638 |
| CREA | 1052 | CREA | 0.0078 | 0.0030 | 1.0078 | 1.0018 | 1.0139 | 0.0102 |
| DIABETES | 1052 | factor(DIABETES)1 | 0.2614 | 0.1781 | 1.2988 | 0.9161 | 1.8414 | 0.1421 |
| GLU | 1052 | GLU | 0.1226 | 0.0453 | 1.1304 | 1.0343 | 1.2355 | 0.0069 |
| HYPERTENSION | 1052 | factor(HYPERTENSION)1 | 0.3739 | 0.1269 | 1.4534 | 1.1334 | 1.8638 | 0.0032 |
| MARITAL recoded | 1052 | factor(MARITAL.1)2 | 0.8392 | 0.3463 | 2.3145 | 1.1741 | 4.5626 | 0.0154 |
|  |  | factor(MARITAL.1)3 | 0.2559 | 0.7004 | 1.2917 | 0.3273 | 5.0976 | 0.7148 |
|  |  | factor(MARITAL.1)4 | 1.1588 | 0.4195 | 3.1861 | 1.4002 | 7.2497 | 0.0057 |
|  |  | factor(MARITAL.1)5 | -11.6170 | 324.7439 | 0.0000 | 0.0000 | inf. | 0.9715 |
| EXERCISE recoded | 1052 | factor(EXERCISE.1)2 | -0.1089 | 0.2171 | 0.8968 | 0.5861 | 1.3723 | 0.6158 |
|  |  | factor(EXERCISE.1)3 | -0.1108 | 0.2110 | 0.8951 | 0.5920 | 1.3535 | 0.5995 |
|  |  | factor(EXERCISE.1)4 | 0.3323 | 0.2434 | 1.3941 | 0.8653 | 2.2462 | 0.1721 |
|  |  | factor(EXERCISE.1)5 | 0.0934 | 0.1716 | 1.0979 | 0.7843 | 1.5370 | 0.5862 |
|  |  | factor(EXERCISE.1)6 | 12.7160 | 324.7437 | 333035.0945 | 0.0000 | inf. | 0.9688 |
| ECONOMIC_4 recoded | 1052 | factor(ECONOMIC.1)2 | -0.0482 | 0.1805 | 0.9530 | 0.6691 | 1.3574 | 0.7896 |
|  |  | factor(ECONOMIC.1)3 | -0.2017 | 0.1812 | 0.8173 | 0.5730 | 1.1657 | 0.2654 |
|  |  | factor(ECONOMIC.1)4 | -0.0338 | 0.1914 | 0.9667 | 0.6643 | 1.4068 | 0.8597 |
|  |  | factor(ECONOMIC.1)5 | -0.1760 | 0.1962 | 0.8386 | 0.5710 | 1.2318 | 0.3696 |
| WC categorical recoded | 1052 | factor(WC.3)1 | 0.4446 | 0.2151 | 1.5599 | 1.0234 | 2.3778 | 0.0387 |
|  |  | factor(WC.3)2 | 0.1732 | 0.2355 | 1.1891 | 0.7495 | 1.8865 | 0.4621 |
|  |  | factor(WC.3)3 | 0.4216 | 0.3425 | 1.5245 | 0.7791 | 2.9831 | 0.2183 |
