# Supplementary material for: The association of nocturnal hypoxemia with dyslipidemia in sleep-disordered breathing population of Chinese community: a cross-sectional study
Source: Lipids Health Dis. 2023 Sep 26;22:159. doi: 10.1186/s12944-023-01919-8 (PMC10521560; doi:10.1186/s12944-023-01919-8)
Supplement: Supplementary file 11 — Additional file 11: Table S6. The change in the regression coefficient of the meanSpO2. [file 12944_2023_1919_MOESM11_ESM.doc]

**Table S6. Introduce covariates in the basic model and eliminate covariates in the complete model to observe the change of the regression coefficient of meanSpO2**

|  |  | basic model | complete model |  |
| --- | --- | --- | --- | --- |
| Covariates | +/- term | MEANSPO2 | MEANSPO2 | Select |
|  | Initial regression coefficient | -0.0912 | -0.0692 |  |
| SEX | factor(SEX) | -0.0845 | -0.0701 |  |
| AGE | AGE | -0.0787 * | -0.0705 | Yes |
| EDU_CATEGORY3 | factor(EDU_CATEGORY3) | -0.0863 | -0.0689 |  |
| SMOKE | factor(SMOKE) | -0.0850 | -0.0696 |  |
| DRINK | factor(DRINK) | -0.0934 | -0.0646 |  |
| AST | AST | -0.0916 | -0.0683 |  |
| CREA | CREA | -0.0855 | -0.0691 |  |
| DIABETES | factor(DIABETES) | -0.0894 | -0.0700 |  |
| GLU | GLU | -0.0859 | -0.0710 |  |
| HYPERTENSION | factor(HYPERTENSION) | -0.0825 | -0.0709 |  |
| MARITAL recoded | factor(MARITAL.1) | -0.0903 | -0.0667 |  |
| EXERCISE recoded | factor(EXERCISE.1) | -0.0907 | -0.0699 |  |
| ECONOMIC_4 recoded | factor(ECONOMIC.1) | -0.0876 | -0.0723 |  |
| WC categorical recoded | factor(WC.3) | -0.0841 | -0.0712 |  |

* indicates a change of more than 10% compared with the initial regression coefficient
