# Supplementary material for: The association of nocturnal hypoxemia with dyslipidemia in sleep-disordered breathing population of Chinese community: a cross-sectional study
Source: Lipids Health Dis. 2023 Sep 26;22:159. doi: 10.1186/s12944-023-01919-8 (PMC10521560; doi:10.1186/s12944-023-01919-8)
Supplement: Supplementary file 12 — Additional file 12: Table S7. Mediation analysis between nocturnal mean oxygen saturation and dyslipidemia in the study population using waist circumference. [file 12944_2023_1919_MOESM12_ESM.doc]

**Table S7. Mediation analysis between nocturnal mean oxygen saturation with dyslipidaemia in the studied population using waist circumference**

|  | Estimate (95% CI) | P-value |
| --- | --- | --- |
| Total effect（MeanSpO2→waist circumference →dyslipidaemia） | -0.031974 (-0.064642,-0.003024) | 0.0260 |
| Mediation effect (MeanSpO2→dyslipidaemia mediated by waist circumference ) | **-0.009805 (-0.018218,-0.003645)** | **0.0020** |
| Direct effect (MeanSpO2→dyslipidaemia) | -0.022330 (-0.057026,0.008210) | 0.1480 |
| Propotion mediated | **0.306647 (0.068930,1.932654)** | **0.0280** |
