# Supplementary material for: The association of nocturnal hypoxemia with dyslipidemia in sleep-disordered breathing population of Chinese community: a cross-sectional study
Source: Lipids Health Dis. 2023 Sep 26;22:159. doi: 10.1186/s12944-023-01919-8 (PMC10521560; doi:10.1186/s12944-023-01919-8)
Supplement: Supplementary file 13 — Additional file 13: Table S8. Baseline characteristics of the participants with and without sleep-disordered breathing. [file 12944_2023_1919_MOESM13_ESM.doc]

**Table S8. Baseline characteristics of participants with and without SDB**

| Characteristics | All Participants  (n=3829) | Without  （n=2651） | Without SDB  （n=1178） | P-value |
| --- | --- | --- | --- | --- |
| Age (years) | 53.904 ± 12.698 | 52.699 ± 12.352 | 56.615 ± 13.048 | <0.001 |
| Sex (%) |  |  |  | <0.001 |
| Female | 1160 (30.295%) | 637 (24.029%) | 523 (44.397%) |  |
| Male | 2669 (69.705%) | 2014 (75.971%) | 655 (55.603%) |  |
| Martial Status (%) |  |  |  | 0.136 |
| Single | 190 (5.108%) | 146 (5.666%) | 44 (3.850%) |  |
| Married | 3251 (87.392%) | 2239 (86.884%) | 1012 (88.539%) |  |
| Divorce | 42 (1.129%) | 30 (1.164%) | 12 (1.050%) |  |
| Widowed | 237 (6.371%) | 162 (6.286%) | 75 (6.562%) |  |
| Education (%) |  |  |  | 0.855 |
| Less than high school | 1770 (47.619%) | 1229 (47.765%) | 541 (47.290%) |  |
| High school | 1059 (28.491%) | 736 (28.605%) | 323 (28.234%) |  |
| More than high school | 888 (23.890%) | 608 (23.630%) | 280 (24.476%) |  |
| Physical exercise (%) |  |  |  | 0.002 |
| 5-7 days per week | 1951 (52.432%) | 1298 (50.349%) | 653 (57.130%) |  |
| 3-4 days per week | 396 (10.642%) | 286 (11.094%) | 110 (9.624%) |  |
| 1-2 days per week | 422 (11.341%) | 305 (11.831%) | 117 (10.236%) |  |
| ≤ 3 days per month | 334 (8.976%) | 253 (9.814%) | 81 (7.087%) |  |
| never exercising | 618 (16.608%) | 436 (16.912%) | 182 (15.923%) |  |
| Cigarette smoking(%) |  |  |  | <0.001 |
| No | 3071 (82.509%) | 2198 (85.260%) | 873 (76.311%) |  |
| Former | 157 (4.218%) | 79 (3.064%) | 78 (6.818%) |  |
| Current | 494 (13.272%) | 301 (11.676%) | 193 (16.871%) |  |
| Alcohol use (%) |  |  |  | <0.001 |
| No | 3107 (83.477%) | 2221 (86.152%) | 886 (77.448%) |  |
| Former | 70 (1.881%) | 42 (1.629%) | 28 (2.448%) |  |
| Current | 545 (14.643%) | 315 (12.219%) | 230 (20.105%) |  |
| Diabetes(%) |  |  |  | 0.074 |
| No | 3209 (86.217%) | 2240 (86.889%) | 969 (84.703%) |  |
| Yes | 513 (13.783%) | 338 (13.111%) | 175 (15.297%) |  |
| Hypertension(%) |  |  |  | <0.001 |
| No | 2543 (68.323%) | 1864 (72.304%) | 679 (59.353%) |  |
| Yes | 1179 (31.677%) | 714 (27.696%) | 465 (40.647%) |  |
| MeanSpO2 | 96.441 ± 1.607 | 96.892 ± 1.121 | 95.424 ± 2.020 | <0.001 |
| MinSpO2 | 86.827 ± 5.265 | 89.034 ± 3.617 | 81.860 ± 5.005 | <0.001 |
| T90% | 6.340 ± 20.696 | 1.322 ± 3.368 | 17.632 ± 34.397 | <0.001 |
| T90(s) | 380.395 ± 1241.756 | 79.339 ± 202.102 | 1057.900 ± 2063.846 | <0.001 |
| Total cholesterol (mg/dL) | 5.608 ± 1.122 | 5.603 ± 1.141 | 5.621 ± 1.079 | 0.655 |
| HDL-C (mg/dL) | 1.413 ± 0.392 | 1.450 ± 0.391 | 1.329 ± 0.381 | <0.001 |
| LDL-C (mg/dL) | 3.242 ± 0.910 | 3.228 ± 0.922 | 3.276 ± 0.882 | 0.139 |
| Triglycerides (mg/dL) | 1.531 ± 1.187 | 1.465 ± 1.144 | 1.678 ± 1.266 | <0.001 |
| AST (U/L) | 24.259 ± 11.734 | 24.19 ± 11.69 | 25.45 ± 9.47 | 0.057 |
| Fasting blood glucose(mmol/L) | 5.932 ± 1.492 | 24.012 ± 12.224 | 24.814 ± 10.533 | 0.055 |
| Creatinine(umol/l) | 70.180 ± 20.554 | 5.890 ± 1.495 | 6.027 ± 1.480 | 0.010 |
| BMI （kg/m²） | 23.598 ± 3.311 | 23.056 ± 3.076 | 24.819 ± 3.493 | <0.001 |
