# Supplementary material for: The association of nocturnal hypoxemia with dyslipidemia in sleep-disordered breathing population of Chinese community: a cross-sectional study
Source: Lipids Health Dis. 2023 Sep 26;22:159. doi: 10.1186/s12944-023-01919-8 (PMC10521560; doi:10.1186/s12944-023-01919-8)
Supplement: Supplementary file 14 — Additional file 14: Table S9. Odds ratios and 95% confidence intervals for dyslipidemia in patients with sleep-disordered breathing. [file 12944_2023_1919_MOESM14_ESM.doc]

Table S9.Odds Ratios and 95% Confidence Intervals of Dyslipidaemia Among Patients with SDB.

|  | Model 1 | Model 2 | Model 3 |
| --- | --- | --- | --- |
| ODI | 1.014 (0.998, 1.030) 0.08258 | 1.009 (0.992, 1.025) 0.30112 | 1.009 (0.992, 1.026) 0.31816 |
| MeanSpO2 | **0.915 (0.860, 0.974) 0.00504** | **0.928 (0.871, 0.989) 0.02051** | **0.929 (0.871, 0.991) 0.02462** |
| MinSpO2 | 0.986 (0.963, 1.011) 0.26674 | 0.993 (0.969, 1.019) 0.60371 | 0.995 (0.970, 1.021) 0.70669 |
| T90% | 1.001 (0.997, 1.004) 0.66450 | 1.000 (0.996, 1.004) 0.96336 | 1.000 (0.997, 1.004) 0.90629 |
| T90 | 1.000 (1.000, 1.000) 0.66450 | 1.000 (1.000, 1.000) 0.96336 | 1.000 (1.000, 1.000) 0.90629 |
| MeanSpO2 quartile |  |  |  |
| Q1 | ref | ref | ref |
| Q2 | 0.857 (0.612, 1.200) 0.36756 | 0.893 (0.630, 1.264) 0.52176 | 0.905 (0.636, 1.288) 0.58017 |
| Q3 | 0.784 (0.559, 1.098) 0.15656 | 0.832 (0.586, 1.180) 0.30146 | 0.869 (0.609, 1.240) 0.43854 |
| Q4 | 0.515 (0.365, 0.726) 0.00015 | 0.569 (0.396, 0.818) 0.00231 | 0.575 (0.397, 0.831) 0.00328 |
| P for trend | 0.001 | 0.004 | 0.007 |

Notes: Model 1: no covariates were adjusted

Model 2: only sociodemographic variables were adjusted (age, sex, education level, marital status)

Model 3: age, sex, education level, marital status,physical exercise,cigarette smoking,alcohol

use ,diabetes,hypertension,AST ,fasting blood glucose,creatinine,**BMI**

Abbreviations: ODI, oxygen desaturation index; MinSpO2, lowest nocturnal oxygen saturation; MeanSpO2, nocturnal mean oxygen saturation; T90, night time spent with an oxygen saturation below 90%; T90%, percentage of night time with oxygen saturation below 90%;BMI,body mass Index.
