# Supplementary material for: The association of nocturnal hypoxemia with dyslipidemia in sleep-disordered breathing population of Chinese community: a cross-sectional study
Source: Lipids Health Dis. 2023 Sep 26;22:159. doi: 10.1186/s12944-023-01919-8 (PMC10521560; doi:10.1186/s12944-023-01919-8)
Supplement: Supplementary file 15 — Additional file 15: Table S10. Association of oxygen desaturation parameters with parameter of the lipid profile. [file 12944_2023_1919_MOESM15_ESM.doc]

Table S10.Association of oxygen desaturation parameters with parameter of the lipid profile

|  | Model 1 | Model 2 | Model 3 |
| --- | --- | --- | --- |
| CHO |  |  |  |
| ODI | -0.003 (-0.012, 0.005) 0.43637 | -0.002 (-0.010, 0.007) 0.70286 | -0.001 (-0.009, 0.008) 0.89404 |
| MeanSpO2 | -0.018 (-0.050, 0.013) 0.25740 | -0.011 (-0.043, 0.021) 0.50923 | -0.011 (-0.044, 0.021) 0.48748 |
| MinSpO2 | 0.003 (-0.010, 0.016) 0.66133 | -0.002 (-0.015, 0.011) 0.78052 | -0.002 (-0.015, 0.011) 0.72221 |
| T90% | -0.000 (-0.002, 0.002) 0.78343 | -0.000 (-0.002, 0.002) 0.89410 | -0.000 (-0.002, 0.002) 0.99027 |
| T90 | -0.000 (-0.000, 0.000) 0.78343 | -0.000 (-0.000, 0.000) 0.89410 | -0.000 (-0.000, 0.000) 0.99027 |
| HDL_C |  |  |  |
| ODI | -0.003 (-0.006, 0.000) 0.05239 | -0.001 (-0.004, 0.002) 0.72440 | -0.000 (-0.003, 0.003) 0.91827 |
| MeanSpO2 | 0.014 (0.002, 0.025) 0.01875 | 0.009 (-0.002, 0.021) 0.09620 | 0.008 (-0.003, 0.020) 0.13576 |
| MinSpO2 | -0.002 (-0.007, 0.003) 0.37604 | -0.005 (-0.009, -0.000) 0.03064 | **-0.006 (-0.010, -0.001) 0.01396** |
| T90% | -0.000 (-0.001, 0.000) 0.36844 | -0.000 (-0.001, 0.001) 0.97330 | -0.000 (-0.001, 0.001) 0.96487 |
| T90 | -0.000 (-0.000, 0.000) 0.36844 | -0.000 (-0.000, 0.000) 0.97330 | -0.000 (-0.000, 0.000) 0.96487 |
| LDL_C |  |  |  |
| ODI | -0.002 (-0.009, 0.005) 0.63506 | -0.002 (-0.009, 0.005) 0.66368 | -0.001 (-0.008, 0.006) 0.80817 |
| MeanSpO2 | -0.019 (-0.045, 0.007) 0.15105 | -0.008 (-0.035, 0.018) 0.52506 | -0.011 (-0.037, 0.016) 0.42626 |
| MinSpO2 | 0.005 (-0.006, 0.015) 0.38926 | 0.002 (-0.009, 0.013) 0.70652 | 0.001 (-0.009, 0.012) 0.80790 |
| T90% | 0.000 (-0.001, 0.002) 0.96137 | -0.000 (-0.002, 0.001) 0.97113 | 0.000 (-0.002, 0.002) 0.98725 |
| T90 | 0.000 (-0.000, 0.000) 0.96137 | -0.000 (-0.000, 0.000) 0.97113 | 0.000 (-0.000, 0.000) 0.98725 |
| TG |  |  |  |
| ODI | 0.002 (-0.008, 0.012) 0.66998 | 0.000 (-0.010, 0.010) 0.95359 | -0.000 (-0.010, 0.009) 0.92560 |
| MeanSpO2 | -0.018 (-0.055, 0.020) 0.35060 | -0.017 (-0.055, 0.021) 0.37902 | -0.011 (-0.048, 0.026) 0.56094 |
| MinSpO2 | 0.001 (-0.014, 0.017) 0.85029 | 0.003 (-0.012, 0.019) 0.66910 | 0.006 (-0.009, 0.021) 0.41131 |
| T90% | -0.000 (-0.003, 0.002) 0.71572 | -0.001 (-0.003, 0.002) 0.59035 | -0.000 (-0.003, 0.002) 0.67287 |
| T90 | -0.000 (-0.000, 0.000) 0.71572 | -0.000 (-0.000, 0.000) 0.59035 | -0.000 (-0.000, 0.000) 0.67287 |

Abbreviations: ODI, oxygen desaturation index; MinSpO2, lowest nocturnal oxygen saturation; MeanSpO2, nocturnal mean oxygen saturation; T90, night time spent with an oxygen saturation below 90%; T90%, percentage of night time with oxygen saturation below 90%,TC,Total cholesterol;TG,Triglyceride;HDL ,High-density lipoprotein;LDL-C,Low-density lipoprotein cholesterol.
