# Supplementary material for: The association of nocturnal hypoxemia with dyslipidemia in sleep-disordered breathing population of Chinese community: a cross-sectional study
Source: Lipids Health Dis. 2023 Sep 26;22:159. doi: 10.1186/s12944-023-01919-8 (PMC10521560; doi:10.1186/s12944-023-01919-8)
Supplement: Supplementary file 16 — Additional file 16: Table S11. Association of minSpO2 with high-density lipoprotein cholesterol. [file 12944_2023_1919_MOESM16_ESM.doc]

Table S11.Association of MinSpO2 with HDL-C

| MinSpO2 quartile | Model 1 | Model 2 | Model 3 |
| --- | --- | --- | --- |
| Q1 | ref | ref | ref |
| Q2 | -0.2 (-0.4, 0.0) 0.097 | -0.2 (-0.4, -0.0) 0.031 | -0.2 (-0.4, -0.0) 0.019 |
| Q3 | 0.0 (-0.1, 0.2) 0.678 | -0.0 (-0.2, 0.2) 0.967 | -0.0 (-0.2, 0.2) 0.948 |
| Q4 | 0.0 (-0.2, 0.2) 0.812 | -0.0 (-0.2, 0.1) 0.647 | -0.1 (-0.2, 0.1) 0.577 |
| P for trend | 0.0 (-0.0, 0.0) 0.582 | -0.0 (-0.0, 0.0) 0.910 | -0.0 (-0.0, 0.0) 0.866 |
